# Chronic cerebral hypoperfusion: a critical feature in unravelling the etiology of vascular cognitive impairment

**DOI:** 10.1186/s40478-023-01590-1

**Published:** 2023-06-12

**Authors:** Vismitha Rajeev, Yuek Ling Chai, Luting Poh, Sharmelee Selvaraji, David Y. Fann, Dong-Gyu Jo, T. Michael De Silva, Grant R. Drummond, Christopher G. Sobey, Thiruma V. Arumugam, Christopher P. Chen, Mitchell K. P. Lai

**Affiliations:** 1 Department of Pharmacology, Yong Loo Lin School of Medicine, National University of Singapore, Singapore, Singapore; 2 Memory Aging and Cognition Centre, National University Health System, Singapore, Singapore; 3 Integrative Sciences and Engineering Programme, NUS Graduate School, National University of Singapore, Singapore, Singapore; 4 School of Pharmacy, Sungkyunkwan University, Suwon, Republic of Korea; 5 Department of Physiology, Anatomy and Microbiology, La Trobe University, Bundoora, Victoria Australia; 6 NUS Healthy Longevity Translational Research Programme, Yong Loo Lin School of Medicine, National University of Singapore, Singapore, Singapore

**Keywords:** Vascular dementia, Neuronal cell death, Chronic cerebral hypoperfusion, White matter lesions

## Abstract

Vascular cognitive impairment (VCI) describes a wide spectrum of cognitive deficits related to cerebrovascular diseases. Although the loss of blood flow to cortical regions critically involved in cognitive processes must feature as the main driver of VCI, the underlying mechanisms and interactions with related disease processes remain to be fully elucidated. Recent clinical studies of cerebral blood flow measurements have supported the role of chronic cerebral hypoperfusion (CCH) as a major driver of the vascular pathology and clinical manifestations of VCI. Here we review the pathophysiological mechanisms as well as neuropathological changes of CCH. Potential interventional strategies for VCI are also reviewed. A deeper understanding of how CCH can lead to accumulation of VCI-associated pathology could potentially pave the way for early detection and development of disease-modifying therapies, thus allowing preventive interventions instead of symptomatic treatments.

## Introduction

**Fig. 1:**
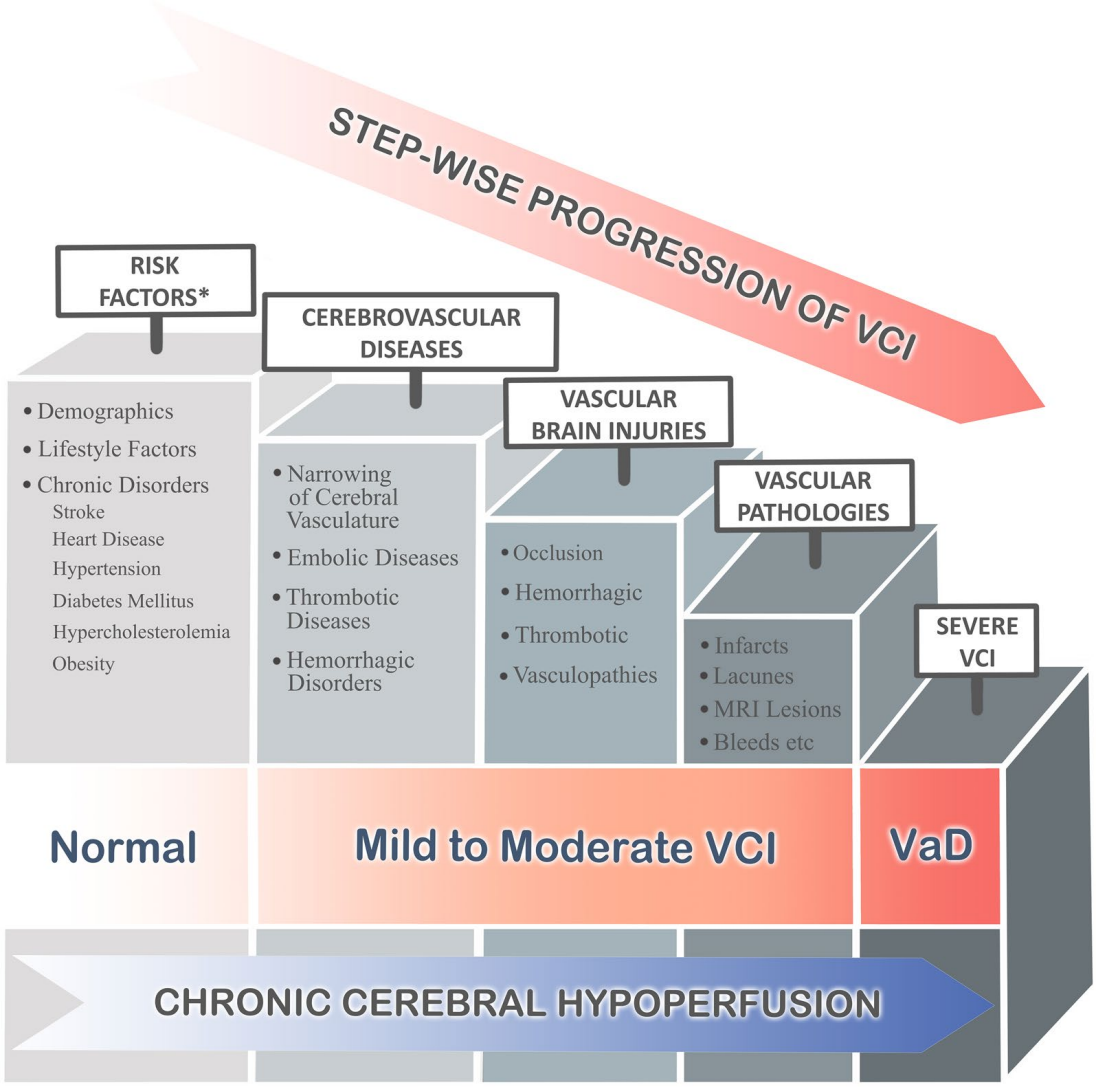
Stepwise progression to VaD. The road from risk factors to disease manifestation in VCI is a complicated one, as the multiple demographics, lifestyle and comorbid disease risk and mitigating factors interact through the progression from asymptomatic vascular lesions, cognitive impairment, and finally to VaD. Furthermore, these complex interactions give rise to several distinct cerebrovascular diseases underlying different forms of vascular brain injuries, leading to the clinical heterogeneity of VaD. However, regardless of the specific nature of vascular injury (occlusive, thrombotic, etc.), a state of chronic cerebral hypoperfusion can be considered to be the common etiological link. *See Table 1 for details and summary of supporting research

A key missing piece in dementia research is the elucidation of the neurovascular basis of cognitive impairment [230, 258, 308]. The term vascular cognitive impairment (VCI) is used to describe a wide spectrum of conditions characterized by cerebrovascular disease ranging from subjective cognitive decline to vascular dementia (VaD) (Fig. 1). While VaD remains the second most common type of dementia worldwide after Alzheimer’s disease (AD), its prevalence may be underestimated—especially in populations with significant concomitant small vessel disease burden, such as those in Asia [42, 48, 49]. Vascular factors may also exacerbate the pathology of AD [221], giving rise to the argument that vascular pathology could even be the most common contributor to dementia in elderly populations [222]. Moreover, VaD is associated with a high mortality rate and rapid stepwise disease progression (Fig. 1) [4, 5, 116]. Therefore, the identification of interventions to potentially benefit VCI patients and reduce its socioeconomic burden is of critical importance.

The pathophysiology and clinical characteristics of VCI have been extensively reviewed [99, 281, 283, 304]. Briefly, VCI is characterized by brain lesions that occur due to vascular pathology which leads to diverse cognitive impairments. These lesions result in ischemic, hemorrhagic, and hypoperfusive states that can manifest as various clinical symptoms. Such vascular pathophysiological states in turn lead to a range of downstream effects and structural changes on the brain, including infarcts, lacunes, microbleeds, white matter injury and parenchymal lesions [108, 139, 140, 147, 228, 247]. VCI thus exists as a heterogeneous group of diseases which can be divided into various subtypes, such as multi-infarct dementia, post-stroke dementia and subcortical ischemic vascular dementia, each having unique features that may manifest clinically as dementia over time. While these disease subtypes provide an avenue to categorize the differing disease etiologies, they have a common set of risk factors, including demographic factors, lifestyle factors or presence of co-morbid conditions (summarized in Table 1). Each of these risk factors is known to independently contribute to the progression of cerebrovascular disease and is therefore associated with cognitive dysfunction and impact the progression to dementia. While there are several treatment options to manage VCI and their underlying risk factors as highlighted in Table 2, the root causes of the problem remain unclear. Hence, there remains several knowledge gaps in the understanding of VCI pathophysiology. Although several mechanisms have been reported to have roles in VCI progression, including neuroinflammation, oxidative stress-induced brain damage, neurodegeneration and brain atrophy [254], we still lack a thorough understanding of this complex disease, and even the aforementioned mechanisms have not been fully elucidated. Furthermore, the clinical signs and symptoms of VCI vary between patients given the heterogeneity of the severity and site of injury. Hence, a consensus on the underlying causes of VCI needs to be reached.

**Table 1:** Risk factors for vascular dementia (VaD)

Risk factors		Description/findings
Demographic	Advanced age	Accounts for many unrecognized vascular changes in the brain. After the age of 65, the risk of developing dementia increases gradually [60]
	Sex/gender	Inconclusive findings
		Some studies report that males are overall at a higher risk till the age of 85 and the overall prevalence of VaD becomes higher in women than in men, especially at very old age (> 85)
		Other studies argue that the protective effects of estrogen in women against coronary heart disease account for a lower risk of VaD in females [60, 94, 98, 251]
	Education	Data is inconclusive but there are studies that report an association between a low formal education with a greater risk of developing VaD [239, 246]
	Social class	Occupational classes such as professional/Intermediate, skilled non-manual, skilled manual and part-skilled/unskilled have shown to be associated with dementia risk. The higher the class, the lower the dementia risks [225, 253]
	Genetic factors	No robust genetic risk factors have been identified. However, *APOE* and *NOTCH3* mutations can be associated with the formation of VCI as these individuals may be predisposed to strokes and other CVD that can potentially manifest as VaD [54, 98, 131, 262, 281]
Lifestyle Factors	Smoking	Smoking and tobacco addiction has been identified as significant risk factors for cardiovascular disease, cerebral vascular disease and cognitive decline. Particularly, smoking causes vascular endothelial dysfunction and atherosclerotic damage [8, 17, 97]
	Cognitive reserve	Cognitive reserve explains the theory that some individuals have a structurally and functionally more resilient brain against injury and disease. This risk factor may be associated with external influences such as education and occupation [69, 225]
	Alcohol use	Heavy drinking or chronic harmful use of alcohol is associated with other vascular risk factors such as high blood pressure, stroke, atrial fibrillation and CHD. Moderate drinking has mostly shown to have beneficial effects, although some studies report structural brain damage [236, 270, 284]
	Diet	Effective individual nutrients such as vitamins E and B can provide for neuroprotective benefits in the brain. Some foods such as saturated fats and trans fats have been shown to increase cognitive decline and hence increase the risk for developing dementia [186–188]
	Physical inactivity	Intervention studies of physical activities on cognition have revealed that indeed the risk of dementia decreases with increased activity. However, there is still insufficient data to confirm this association because increased physical activities complement other risk factors such as risk of obesity and stroke [1, 58]
	Homocysteine	Hyperhomocysteinemia has been shown in studies to be associated with vascular disease. Homocysteine induces cellular damage via oxidative stress, excitotoxicity, and damage to the blood–brain barrier. Studies have also shown an association between high levels of Homocysteine and increased risk of atherosclerosis, atrophy and white matter diseases [3, 109, 211, 256, 274]
Chronic disorders	Stroke	A person with the history of stroke becomes approximately three to nine times as likely to develop VaD as compared to a healthy individual. Furthermore, the risk of VaD increases further in patients who already are suffering from pre-stroke cognitive decline [61, 117, 153, 164, 208]
	CAD/CHD/ischemic heart disease	CAD/CHD/Ischemic Heart Disease has been identified to be a significant independent risk factor for vascular dementia and risk of cognitive decline. Atherosclerosis plays a major role in the development of CAD/CHD and has been observed clinically in many VaD patients [90, 101, 137, 154, 193, 202, 219]
	PAD/PVD	Peripheral arterial disease (PAD) is a manifestation of systemic atherosclerosis in the body and has been reported to increase the risk of dementia types such as AD and VaD by double. This is especially apparent in patients with severe peripheral vascular disease (PVD) and ischemic heart disease. In fact, PAD was associated with a faster cognitive decline independently of previous CVD risk factors [205, 212, 267]
	Atrial fibrillation	This form of cardiac arrhythmia has been shown to be a significant independent risk factor for vascular dementia and AD. Moreover, patients with underlying microvascular dysfunction in addition to AF may manifest VaD earlier [36, 137]
	Hypertension	High blood pressure is not just a risk factor for dementia, but for other conditions such as stroke as well. In fact, many studies have reported that hypertension is an independent risk factor for VaD [137, 190, 303]
	Diabetes mellitus	Studies have reported associations between diabetes and developing early-stage cognitive impairment and also in VaD. Diabetes is also strongly associated with cerebral Vasculopathy. It has been reported that the risk of developing VaD is higher when diabetes occurs at the mid-life stage rather than the late-life stage as other environmental factors provide for a stronger link at the later life stage [111, 137, 204, 216, 302]
	Myocardial infarction	Patients with MI have a higher risk of developing cognitive impairment due to brain hypoperfusion. It has been reported that women with MI are five times more likely to develop cognitive impairment as compared to men. An effect of MI is low cardiac output, promotes brain hypoperfusion and hence is associated with cognitive decline and manifestation into dementia [13, 30, 66, 137, 316]
	Hypercholesterolemia	High cholesterol is one of the risk factors for VaD. Hypercholesterolemia has been shows to be one of the dominant mechanisms in atherosclerosis and hence cognitive decline [9, 66, 75, 209]
	Depression	Although inconclusive, there are studies that report mid- and late- life depression is associated with a higher risk of VaD. Particularly, depression that only begins at the late- life stage is associated with AD, but recurring depression is associated with VaD [21, 37]
	Overweight/obese	Obesity decreases blood supply to the brain and fat cells damage the cerebral white matter leading to cognitive decline and hence VaD. Damaged white matter decreases neuronal functioning and eventual brain atrophy. The mechanism for obesity-induced damage is the obesity-induced release of adipocyte-secreted proteins and obesity-induced inflammatory cytokine release [7, 12, 148]

**Table 2:** Current pharmacotherapeutic options for vascular dementia (VaD)

Drug classification	Drug(s)	Target	Mechanism of action and side effects
Angiotensin Inhibiting Enzyme (ACE) Inhibitors	Enalapril, Lisinopril, Perindopril, Ramipril	Lowers blood pressure	ACE inhibitors inhibit the production of angiotensin II, a vasoconstrictor. Blood vessels are relaxed and raise blood flow
			*Side effects* Persistent dry cough, headaches, dizziness, rashes, hyperkalemia, fatigue, loss of taste [226]
Angiotensin-2 receptor blockers (ARBs)	Candesartan, Irbesartan, Losartan, Valsartan, Olmesartan	Lowers blood pressure	Prevents angiotensin II from binding to its receptors on muscle cells of blood vessels, thus blocking vascular smooth muscle constriction
			*Side effects* Dizziness, headaches, cold or flu-like symptoms [22]
Calcium Channel Blockers	Amlodipine, Felodipine, Nifedipine, Diltiazem, Verapamil	Lowers blood pressure	Prevents Ca^2+^ from entering heart muscle cells and endothelial cells of blood vessels, thus reducing the heart rate and reducing depolarization-mediated contraction of the blood vessels; The combination of reduced heart rate and dilated blood vessels lowers blood pressure
			*Side effects* Headaches, swollen ankles, constipation [244]
Diuretics	Indapamide, Bendroflumethiazide	Lowers blood pressure	Decrease blood volume and venous pressure by promoting urine production and output by the kidneys
			*Side effects* Dizziness, headaches, dehydration, rash, muscle cramps, low blood potassium and sodium levels, gout, increased cholesterol [152]
Beta Blockers	Atenolol, Bisoprolol	Lowers blood pressure	Beta blockers interfere and block the binding of epinephrine/adrenaline on beta receptor sites found on the heart. Beta blockers slow down the heart rate and eases the contraction of the heart
			*Side effects* Dizziness, headaches, tiredness, cold hands and feet [295]
Statins	–	Lowers cholesterol	Reduces cholesterol biosynthesis in the liver by inhibiting HMG-CoA reductase
			*Side effects* Diarrhea, headache [291]
Low-dose Aspirin	–	Anti-platelet drug/Reduces the risk of blood clots/Blood thinning	Aspirin inhibits the production of enzyme Cox-1 which produces thromboxane A-2. Thromboxane A-2 is required for platelet aggregation
			*Side effects* Mild indigestion, increased bleeding, allergy.[142, 182]
Clopidogrel	–	Anti-platelet drug/Reduces the risk of blood clots/Blood thinning	Inhibits the P2Y receptor, which is involved in the platelet activation and cross-linking of fibrin
			*Side effects* Increased bleeding, diarrhea, stomach pain, indigestion, heartburn [134]
Warfarin	–	Anti-coagulant drug	Warfarin inhibits the vitamin K dependent synthesis of clotting factors in the blood
			*Side effects* Increased bleeding, stomachache [113]
Metformin	–	Controls blood sugar	Increases body’s sensitivity to insulin and lowers blood sugar levels
			*Side effects* Stomachache, diarrhea, nausea [129]
Antipsychotic Drugs	Haloperidol, Risperidone, Quetiapine	Treats behavioral and psychological symptoms	Dampens emotional behavior by blocking dopamine D2 receptors in the brain
			*Side effects* Anti-cholinergic effect, sedation, weight gain, erectile dysfunction in males [261]

Chronic cerebral hypoperfusion (CCH) refers to chronically inadequate brain perfusion [56]. Given that CCH is intimately associated with various risk factors, pathophysiological processes and pathological lesions known to be involved in VCI (Fig. 1), we propose that CCH is the central underlying cause for the progression of VCI. We emphasize CCH as the underlying cause as it ties together some of the known mechanisms of VCI such as chronic inflammation, oxidative stress, neurodegeneration and brain atrophy. Age-related vascular changes lead to a state of global CCH and induce pathophysiological changes such as blood–brain barrier dysfunction, resulting in increased vulnerability to disease even in the absence of risk factors [278]. Recently, CCH was identified as the common feature observed in multiple subtypes of VCI [74, 283]. Furthermore, it was reported that global cerebral blood flow was significantly lower in VaD than in age-matched controls [227, 231] but significantly higher than in AD [227]. The reductions in cerebral blood flow is one of the earliest features observed from early VCI to VaD [130, 145, 224], and is consistently observed in different brain regions, such as a reported 31% decrease in cerebral blood flow in the frontal cortex and a 39% decrease in the parietal cortex [234]. Compromised cerebral blood flow in the deep white matter of the brain is also associated with hemodynamic ischemic injury, and therefore leads to a higher volume of white matter lesions (WMLs) [18, 286]. Moreover, cerebral hypoperfusion was shown to be a good predictor for WMLs in VCI patients [176, 198]. Given the above evidence, we postulate that CCH is a common driver of VCI pathologies such as WMLs, lacunes, infarcts, and subsequent cognitive impairment.

CCH is strongly associated with stepwise cognitive decline in VCI, where much of what is known about its clinical manifestation along the spectrum from normal to end-stage VCI comes from multiple longitudinal studies involving recruited subjects [128, 135, 200, 224, 268, 275, 286]. Neuropathological evidence, neuropsychological assessments and imaging are important adjuncts in many of these studies to ensure accurate study recruitment. In this review, we explore the concept that CCH is the main mediator of VCI pathology and cognitive impairment. We highlight the links between mechanisms and the development of structural neuropathological changes during VCI and provide a landscape of how each of these changes lead to the development of cognitive impairment in patients. Understanding how CCH drives VCI progression from pre-clinical to severe dementia is essential for both researchers and clinicians in diagnosing and developing novel therapeutics for early intervention.

## Pathophysiology of chronic cerebral hypoperfusion (CCH)

**Fig. 2:**
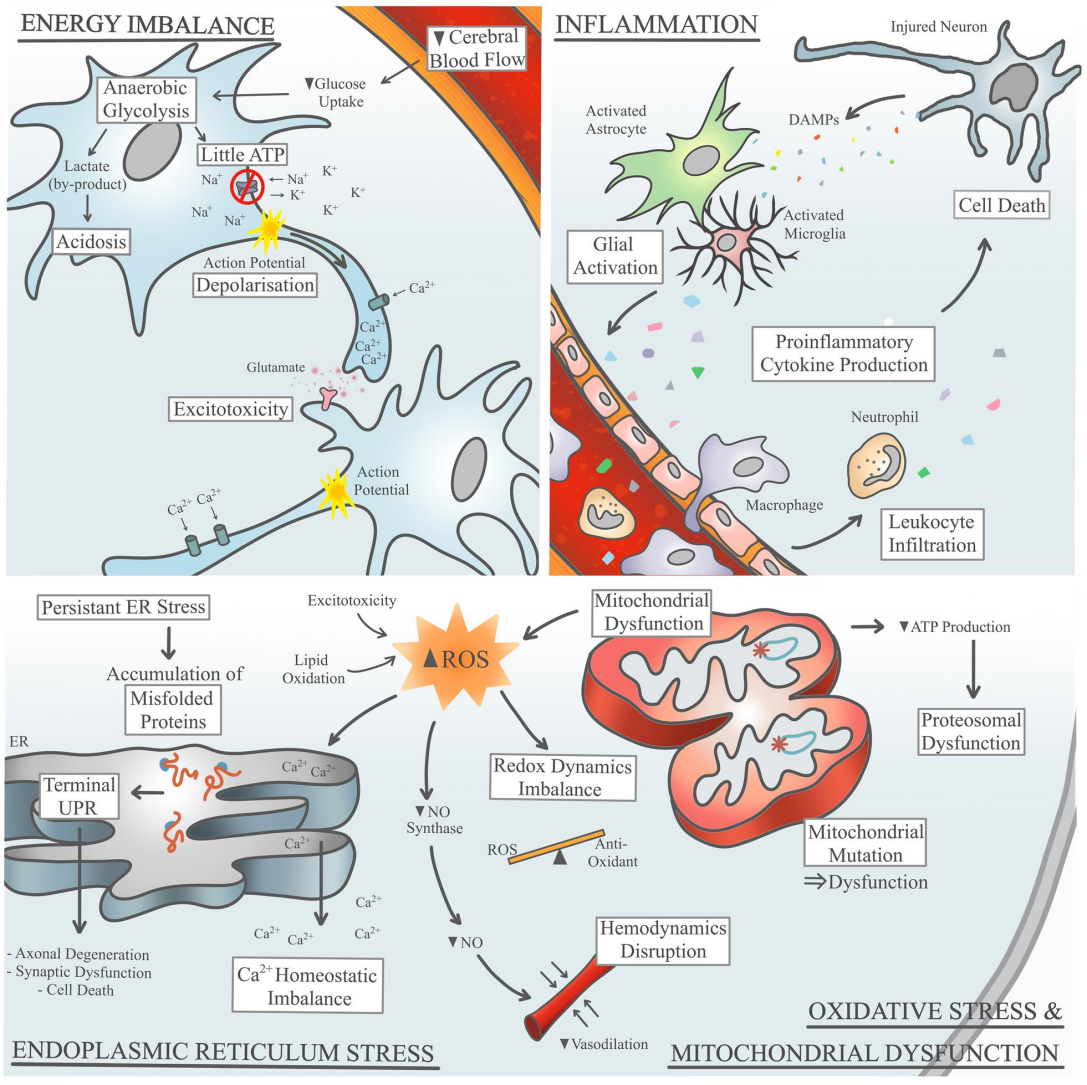
Pathological drivers of CCH-associated VCI. Several CCH-induced pathological drivers have been long associated with the pathogenesis of VaD including energy imbalance, inflammation, endoplasmic reticulum (ER) stress, oxidative stress and mitochondrial dysfunction. Decreased ATP production impairs ATPase pumps, results in neuronal depolarization, and leads to a deregulation in the glutamate homeostasis at the synaptic cleft and excitotoxicity in the brain. Low cerebral blood flow triggers the brain to utilize anaerobic respiration to produce ATP and this results in the accumulation of lactate within the neurons, leading to acidosis. Neuronal death following CCH is primarily attributed to the increase in the pro-inflammatory cytokine release during the chronic inflammatory response. Danger associated molecular patterns (DAMPs) released by the brain cells can also trigger glial activation and leukocyte infiltration, both of which can also produce pro-inflammatory cytokines. At the cellular level, increase in the reactive oxidative species from various sources, including the mitochondria, induces the oxidative stress state. While the increase in reactive oxygen species can contribute to the redox dynamics and hemodynamics imbalance, it can also induce chronic ER stress. Persistent ER stress leads to an accumulation of misfolded proteins and can have fatal effects on neuronal survival and integrity via the terminal unfolded protein response (UPR) pathway, as well as contributing to Ca^2+^ homeostatic imbalance. Mitochondrial deterioration causes a further decrease in the ATP production, leading to proteosomal dysfunction, as well as contributing to the frequency of mutagenesis events at the mitochondrial DNA. In a chronic state of CCH, these drivers are pathological and ultimately pave the way for downstream disease mechanisms

In VCI, isolated instances of vascular injury may accumulate into widespread damage that overcome intrinsic repair mechanisms in areas critical for cognitive functions. There are several possible mechanisms that govern the transition from a physiological to a pathological state in the brain during cerebral vascular disease. In order to understand how this transition occurs, animal studies employing CCH are commonly used to model the underlying pathology of VCI. Mouse CCH models are generally generated by manipulating the common carotid artery, for instance, via bilateral common carotid artery stenosis [242] or asymmetric common carotid artery surgery [114], both of which could lead to reduced cerebral blood flow, white matter rarefaction, glial activation, as well as subsequent cognitive impairment. A cascade of molecular and cellular events has been shown to be involved in the pathogenesis of CCH including energy imbalance, oxidative stress, endoplasmic reticulum stress, mitochondrial dysfunction and inflammation (Fig. 2).

### Pathogenic mechanisms

#### Energy imbalance

During VCI, blood vessels in the brain including arterioles, veins and capillaries are partially occluded or hypoperfused [34, 145, 234]. Disruption to glucose and oxygen supply compromises the production of adenosine triphosphate (ATP) [118, 119]. The resultant state of energy imbalance impairs the function of ATP-dependent sodium–potassium pumps [82] which are critical for maintaining the resting membrane potential of neurons. As such, neurons spontaneously depolarize and release the excitatory neurotransmitter glutamate into the synaptic cleft. The excess accumulation of glutamate in the synaptic cleft is exacerbated by other defective ion pumps that fail to recycle the glutamate, leading to persistent depolarization and overstimulation of neighboring neurons [14, 38]. This excessive activation of glutamate receptors (i.e. NMDA and AMPA receptors) due to energy imbalance that results in neuronal dysfunction and death is called excitotoxicity, which has been reported to occur in chronic diseases such as VCI [265]. In order to compensate for the lack of glucose, the brain will begin to undergo anaerobic glycolysis, which produces lactate. Accumulation of lactate in the brain in turn leads to acidosis and acidotoxicity [143, 301].

#### Oxidative stress

Oxidative stress is defined as an environment where pro-oxidant species dominates over anti-oxidant species [95]. It is one of the central drivers of pathology in many diseases and it is implicated in the cognitive decline in VCI [26, 53, 166, 172]. Correspondingly, a reduction of circulatory antioxidant enzyme levels (e.g., superoxide dismutase, catalase) and antioxidant capacity (e.g., glutathione, ergothioneine) have been observed in VCI patients [80, 241, 297, 298]. In the brain, CCH causes a disruption in calcium (Ca^2+^) homeostasis which leads to acute and chronic production of reactive oxygen species [82, 166] from various sources, including electron transport chain, nicotinamide adenine dinucleotide phosphate oxidases (Nox) and nitric oxide synthase. In animal models of CCH, reduction of endothelial nitric oxide synthase expression [189] disrupts the vascular tone and exacerbates cerebral blood flow hypoperfusion [83]. CCH also increases Nox-1 expression in neurons, inducing apoptosis and contributing to cognitive impairment [53]. Oxidative stress also increases levels of circulating nitric oxide synthase inhibitor, reducing nitric oxide bioavailability, leading to vasodilation impairment as evident in cognitive impairment [67]. As the stiffness and pulsatility of the vessels increase, higher sheer stress is generated which disrupts normal continuous blood flow [19]. These vascular changes have been reported to be associated with reduced blood supply to white matter regions, thus precipitating the formation of white matter lesions and lacunes [266, 293].

#### Endoplasmic reticulum stress

Endoplasmic reticulum stress is emerging as a pathological mechanism in the etiology of VCI [195]. The endoplasmic reticulum is involved in the synthesis and post-translational modifications of molecules that are important in maintaining Ca^2+^ homeostasis [300]. Being the site of translation, protein folding and transport, disruptions to endoplasmic reticulum’s physiological function in the form of endoplasmic reticulum-calcium depletion, hypoxic conditions and oxidative stress, are known to result in misfolding and accumulation of unfolded integral proteins. Such stressors to endoplasmic reticulum activate an adaptive stress response pathway known as the unfolded protein response (UPR) [229]. This pathway involves three independent endoplasmic reticulum membrane-associated sensors which are protein kinase R-like endoplasmic reticulum kinase (PERK), inositol-requiring protein 1 (IRE1) and activating transcription factor 6 (ATF6) [300].

Under prolonged endoplasmic reticulum stress, cellular proteostasis becomes unsustainable, resulting in accumulation of misfolded proteins and activation of terminal UPR [120]. Attenuation of endoplasmic reticulum stress-induced apoptosis has been found to confer protection against ischemia and reperfusion injury [296]. More specifically, studies using neuronal models of vascular dementia have shown the contribution of zinc-induced neurotoxicity to its pathogenesis, upregulating endoplasmic reticulum stress-related genes like CCAAT-enhancer-binding protein homologous protein (CHOP) and growth-arrest- and DNA-damage-inducible gene 34 (GADD34) [263]. The same group also found that the endoplasmic reticulum stress pathway is involved in zinc-induced neurotoxicity thus implying its possible roles as both a cause and consequence in driving VaD [144].

#### Mitochondrial dysfunction

Given the high energy demands of the brain, mitochondria, as the ‘powerhouse of the cell’, play a central role in producing energy in the form of ATP. Mitochondria are also vital in regulating brain cell survival and death by controlling the movement of calcium ions between the cells and the extracellular surroundings. Reactive oxygen species produced by the mitochondrial energy-redox axis can signal for apoptosis when the cells are damaged [104, 159, 196, 218].

Mitochondrial dysfunction results in decreased energy production, thus altering cellular redox dynamics in the brain. Under these conditions, mitochondria begin producing an excess of ${\text{O}}_{2}^{ \cdot - }$ and H_2_O_2_ molecules in response to increased oxidation of proteins, phospholipids and DNA that pushes the redox equilibrium towards a pro-oxidative state [192]. There is also a global reduction in mitochondrial protein complexes over time [104, 159, 161, 310]. Therefore, it is not surprising that mitochondrial dysfunction has been observed in VaD [162]. In particular, mitochondrial damage such as increased mitochondrial bioenergetic deficits in the hippocampus plays important roles in the spatial learning and memory decline in both human patients and in CCH rodent models [16, 72, 162, 172]. Defects in mitochondrial metabolism lead to altered patterns in the mitochondrial respiratory rate, altered membrane potential, decreased pyruvate hydrogenase levels, increased oxidative stress as manifested by increased hydrogen peroxidase levels. However, mitochondrial dysfunction may not occur independently of other pathological processes but is commonly observed to overlap with other mechanisms such as oxidative stress and proteasome dysfunction [70].

Mitochondrial DNA contains genes that encode the cell’s mitochondrial energy production machinery, and defects or mutations in the mitochondrial DNA have been associated with age-related dementia and neuropathology [29, 31, 68, 102, 287]. The m.3316G > A mutation has been identified in early-onset VaD patients who did not manifest typical vascular symptoms [155]. Rather, the mutation causes a reduction in the activity of the respiratory chain complex I, and hence is associated with the well-established link that cerebrovascular damage increases when mitochondrial energy chain complexes are compromised [125].

#### Neuroinflammation

Inflammation involves a complex range of responses that are known to play a role in disease conditions. While inflammation is important in tissue repair and recovery, under disease conditions, chronic activation of inflammatory responses results in a destructive phenotype that is observed during disease development and progression [62]. Both acute and chronic inflammation have been implicated in cellular injury associated with a hypoxic state of VCI [136, 174, 217]. Under CCH, reduced blood supply disturbs cellular integrity, activates glial cells and recruits peripheral immune cells to the brain [23, 141, 309], causing death of neighboring cells and secondary tissue damage. Various molecular mechanisms such as activation of inflammatory pathways and inflammasome activation have been shown to play a role in inflammation during CCH.

Systemic inflammation serves as the initial signal of a stressed cell involving the release of damage associated molecular patterns (DAMPs), which are recognized by the pattern recognition receptors, namely Toll-like receptors (TLR) and NOD-like receptors (NLR) on neighboring cells, initiating an inflammatory response [82, 260]. Further studies have established the association of NLR family pyrin domain containing 3 (NLRP3) and Absent in melanoma-2 (AIM2), both of which are involved in inflammasome activation, with VaD and CCH [81, 177, 206, 207]. Regulatory pathways such as nuclear factor kappa B (NF-κB) and mitogen-activated protein kinase (MAPK) are subsequently activated to upregulate a wide range of inflammatory proteins [105, 146] including interleukin (IL)-1β, IL-6 and tumor necrosis factor (TNF) [24, 317]. These inflammatory cytokines can cause cell death, oligodendrocyte damage and demyelination [264, 305]. Attenuation of IL-1β production was shown to ameliorate hypoperfusion-induced brain injury in mice [206]. Microglia and astrocytes also release adhesion molecules and chemokines, which activate and facilitate leukocyte infiltration [15, 20, 115]. In a mouse model of cerebral ischemia, genetic deletion of the chemokine CCL2 has been shown to reduce brain injury via modulation of inflammation. Other proinflammatory proteins such as c-reactive protein are also upregulated to facilitate cerebral inflammation in VCI patients [77, 215, 232].

The complement system has also been implicated in stroke [168]. Complement proteins promote inflammation via glial activation and induce neuronal injury through the C5 activating membrane attack complex (MAC). Formation and deposition of C5b-9/MAC complexes damages the myelin sheath [175, 233], and abrogation of C5 protein reduces glial activation and white matter ischemia under CCH [167]. The central effector protein in the system is the C3 convertase enzyme complex [28, 197, 240]. It has been demonstrated that under CCH, microglial cells aggravate white matter injury via the C3-C3aR pathway in rat brains [311]. Together, the entire inflammatory process facilitates astrogliosis and scar formation, oligodendrocyte and endothelial cell dysfunction and blood–brain barrier disruption [248, 282, 313], leading to neurodegeneration, neurovascular dissociation and eventually structural damage to the brain. Therefore, inflammation serves as a critical mechanism that drives subsequent pathological changes in CCH. In summary, the pathological mechanisms of CCH covered in the above section lay a foundation to comprehend the complex mechanistic underpinnings of the disease. The pathological mechanisms of CCH include the involvement of multiple molecules and signaling pathways, especially those related to inflammation and oxidative stress.

### Neuropathological features of CCH

In this section, we will examine how the pathogenic mechanisms described above contribute to the neuropathological features that have been described in VCI.

#### Glial activation

The term neurovascular unit describes the structural and functional interactions between neurons, glial cells, pericytes, extracellular matrix components and endothelial cells in the brain. The neurovascular unit maintains homeostasis within the brain microenvironment ensuring optimal conditions for function of neurons and other cells. During CCH, the entire neurovascular unit is affected by the combined effects of the pathological mechanisms described above that can cause reduced integrity of the neurovascular unit [238]. This results in a homeostatic imbalance in the brain.

Glial cells, especially microglia, drive inflammatory responses by releasing proinflammatory molecules. Increased number of glial cells are commonly seen in VCI patients especially at the white matter regions [245, 269]. Mechanistic studies using animal models have shown that upon CCH, activated microglial cells participated in both systemic and complement-activated inflammation; whereas attenuation of microglial activity reduces proinflammatory cytokine levels, increases myelin density and eventually improves cognitive performance [138, 311].

Astrocytes are also involved in the process of inflammation during CCH. Astrogliosis has direct influence on blood–brain barrier integrity and induces damage when constitutively activated astrocytes form glial scars or swelling at the end feet processes [88, 211]. With CCH, a study reported decreased astrocyte polarity and structural support to the endothelial cells eventually contributing to blood–brain barrier damage [127].

In the white matter, oligodendrocytes are the predominant glial cell type, and produce the myelin sheath around myelinated axons. As CCH damages oligodendrocytes and white matter, repair mechanisms are often impaired due to inflammation and loss of growth factors released by neurons, microglia and astrocytes. The myelin-independent axonal support from oligodendrocyte is also affected, causing significant axonal loss [93, 181, 313]. Upon ischemia, oligodendrocytes also release inhibitory proteins Nogo-A and MMP-9, preventing neuronal remodeling, and initiating a deleterious cascade within white matter to cause blood–brain barrier damage [93, 181].

Together, dysfunction in each of the components within the neurovascular unit can result in the disruption of brain homeostasis, which can eventually lead to neuronal loss and white matter infarctions at the grey matter and the deep white matter territory [35, 252].

#### Activation of cell death

Programmed cell death is a critical role in animal development and tissue homeostasis. Abnormal regulation of programmed cell death is associated with various human diseases including neurodegeneration. Different forms of cell death such as apoptosis, pyroptosis and autophagy have been observed in cerebral ischemia and reperfusion injury [76, 84, 141]. Of these, there has been ample evidence in the literature implicating apoptosis in VCI. In postmortem studies of VCI patients, apoptotic vascular cells were identified in the basal ganglia and subcortical white matter regions [103]. Apoptotic neuronal cells were also observed at cortical layers 3 and 5; and extensive ischemic lesions and axonal damage were observed in severe dementia [103]. Furthermore, protein expression and proteomics studies have revealed a decreasing anti-apoptotic proteins expression pattern in the cortex of VCI patients compared to controls [64]. Within regions of leukoaraiosis, significant increases in apoptotic oligodendrocytes were observed compared to adjacent white matter [33]. Mirroring the evidence seen in human patients, animal models of CCH present similar results, with increased markers of apoptosis observed. Specific changes observed in these animal studies include increased visualization of apoptotic bodies, increased expression of apoptotic proteins such as caspase 3, and reduced expression of anti-apoptotic proteins such as Bcl-2 [194, 243, 257, 272, 290].

More recently, other forms of cell death such as pyroptosis have also been investigated in human patients [24] as well as rodent models of CCH [206, 312]. Autophagy has gained interest as well, having been shown to be upregulated specifically in VaD [41] and in CCH rodent models [47, 51, 126, 306].

These findings, in relation to cell death mechanisms being implicated in the pathophysiology of CCH, reinforce the concept of degeneration over the course of VCI progression, and may suggest that therapeutic interventions in cell death pathways may prove effective in curbing the pathological progression of VCI.

#### Blood–brain barrier dysfunction

The blood–brain barrier is a selectively permeable barrier that separates the circulating blood from the parenchymal tissue. The endothelial cells of the BBB are characterized by expression of tight junction proteins between adjacent cells, reduced rate of transcytosis and other transcellular movement across the barrier into or out of the brain. This property of blood–brain barrier establishes a finely tuned microenvironment for the brain by maintaining homeostasis and defending against pathogenic infections. In CCH, increased blood–brain barrier permeability has been observed [40, 191, 214, 277, 292], and is associated with neuronal loss and white matter degeneration during disease progression [271]. Blood–brain barrier damage can be induced through increased excitotoxicity, inflammation and oxidative stress, which can contribute to further brain injury via mechanisms such as increased leukocyte infiltration.

Excitotoxicity causes a persistent activation of endothelial cells causing cell death and uncontrolled movement of substance across the blood–brain barrier [6, 59]. Separately, high Ca^2+^ levels in the cytosol of the endothelial cells can also lead to activation of cell death mechanisms and the increased propagation of Ca^2+^ levels through the intracellular sources of Ca^2+^ such as mitochondria, and endoplasmic reticulum [92] can relocate the endothelial tight junction proteins [32]. Presence of proinflammatory cytokines can directly damage the blood–brain barrier, reducing its integrity by inducing endocytosis of the tight junction proteins thereby weakening the tight junction assembly [96, 307]. The internalised tight junction proteins are directed to lysosomal degradation, leading to long-term blood–brain barrier dysfunction [250, 279]. Reactive oxygen species have also been implicated in the progression of VaD and could possibly contribute to the breakdown of the blood–brain barrier [211]. Increased reactive oxygen species in endothelial cells downregulates epithelial cadherin levels [2] and bioavailability of nitric oxide, leading to endothelial and blood–brain barrier dysfunction [55, 89].

Blood–brain barrier dysfunction, oxidative stress [112] and inflammation induce matrix metalloproteinases (MMPs)-mediated proteolytic degradation of the extracellular matrix [63, 223, 250]. In both VaD and experimental CCH, increased levels of gelatinases (MMP-2 and MMP-9) have been reported [46, 223], which are associated with the degradation of basement membrane and tight junction proteins of the blood–brain barrier [160, 276]. Blood–brain barrier damage also increased the size of the perivascular spaces leading to cellular damage of pericytes within, a common observation in VaD. Association of pericyte damage with CCH, white matter damage, neuronal loss and cognitive impairment is evident [185] although a direct link between pericytes to VaD has yet to be demonstrated.

The blood–brain barrier regulates immune cell infiltration by maintaining low levels of leukocyte adhesion molecules on endothelial cells with inhibitory effects derived from pericytes [289]. In VaD, inflammation increases expression of adhesion molecules and chemokines such as intercellular adhesion molecule-1 (ICAM-1) and vascular adhesion molecule (VCAM) in endothelial cells [259], facilitating leukocyte infiltration [165, 171]. Upon crossing the damaged blood–brain barrier, activated leukocytes cause irreversible damage to the blood–brain barrier and contribute to further release of pro-inflammatory cytokines and reactive oxygen species, which forms a vicious feedback loop of activating endothelia [50]. Although increased ICAM levels have been reported in post-mortem studies of VaD patients [180], evidence showing specific temporal dynamics of leukocyte movement into the brain is still lacking. Overall, the establishment of endothelial cell activation upon CCH not just damages the blood–brain barrier, it also causes a reduction in the resting cerebral blood flow, and thus further contributes to a hypoperfused state within the brain. The biggest challenge here is the myriad aspects of blood–brain barrier damage and its downstream mechanisms in the context of VCI and other neuropathologies that remain unknown.

#### White matter lesions

One of the major pathological hallmarks of VCI is the formation of white matter lesions (WMLs) [18, 286]. The white matter functions to connect and preserve neural circuit signaling, thus implying the clinical importance of WMLs as markers of brain dysfunction due to cerebral vessel disease. Pathologically WMLs represent processes ranging from demyelination, astrogliosis, axonal loss and venular damage. These are in turn a consequence of the combined effects of increased oxidative stress and inflammation in the brain induced by CCH and blood–brain barrier breakdown [150, 163]. Our group has reported that disruption to the structural integrity of white matter can cause cognitive dysfunction [107, 123]. Anatomically, the white matter region comprises of numerous nerve fiber tracts that are surrounded by myelin. During disease progression, demyelination may occur due to various reasons. Excitotoxicity, oxidative stress and inflammation lead to oligodendrocyte damage through the loss of cellular function, mitochondrial dysfunction and production of pro-apoptotic signaling proteins, eventually contributing to their death and white matter injury [179, 255], and causing primary or secondary myelin destruction in white matter regions [178].

There is limited evidence regarding remyelination at the site of white matter injury following CCH. Remyelination is uncommon during CCH as the chronic hypoxic and pro-oxidative states block the ability of oligodendrocyte progenitor cells from being able to differentiate into newly matured oligodendrocytes [86, 91], a process further impeded by surrounding damaged endothelial cells and scar-formation during astrogliosis [10]. The age-dependent Wnt signaling pathway, which plays a role in the oligodendrocyte progenitor cells differentiation, is also compromised in VCI disease states leading to further remyelination dysfunction [130]. Nevertheless, despite being vulnerable to vascular injury, evidence showed restoration of oligodendrocyte progenitor cells and oligodendrocytes after prolonged CCH (i.e. 1 month of bilateral common carotid artery stenosis), suggesting their potential regeneration ability [181]. Studies suggested that oligodendrogenesis and regeneration are facilitated by reactive astrocytes, which secrete trophic factors such as brain-derived neurotrophic factor in response to white matter injury [169, 183, 184].

## Epigenetic and genetic mechanisms

While VCI progression is mostly sporadic in nature, some forms of VCI are known to be influenced by the interplay of genetics and epigenetics. With technological advancements, researchers have improved access to diagnostic tools which carry out high throughput genomic-based investigations. The following section highlights recent research in the genetic factors of VCI as well as emerging interest in the epigenetics of VCI progression.

### Epigenetics

Epigenetics refers to the alteration of gene expression without altering corresponding DNA sequences [170]. Epigenetic mechanisms are driven primarily by environmental stimuli including stress, diet and other behavioural factors. Given that most of the risk factors of VaD are associated with lifestyle-associated conditions such as hypertension and diabetes mellitus, the role of epigenetics seems to be critical in explaining the pathophysiology of the disease [203]. In fact, there are several lines of evidence for epigenetic contribution in the pathophysiology of dementia in general. These include studies where DNA methylation and hydroxymethylation were observed to be significantly reduced in the hippocampus, entorhinal cortex, cerebellum, and prefrontal cortex of AD patients compared to healthy controls [170]. Epigenetic modifications in AD neuropathology have been increasingly studied with the findings also implicated to other neurodegenerative diseases [79]. While there is limited evidence for the role of epigenetics specifically in VCI or CCH [203, 237, 299], this is likely due to the nascent nature of this topic, pointing to the need for further studies. Nevertheless, with epigenetic changes seen as drivers of pathological conditions, they may be regarded as biomarkers for early disease detection [199]. As such, further study of epigenetics would provide insights into VCI and perhaps aid in the stratification within VCI.

### Genetic mutations

Certain forms of VCI onset and progression are known to have a familial component though the majority are sporadic cases [173]. Monogenic influences of tissue responses to VCI include *NOTCH3* mutations causing cerebral autosomal dominant arteriopathy with subcortical infarcts and leukoencephalopathy (CADASIL), a rare form of cerebrovascular disease. The Notch pathway is important in the regulation of cell fate [121]. In particular, the Notch 3 receptor-mediated pathway is involved in the vascular smooth muscle survival [173]. In CADASIL, the mutation of the *NOTCH3* gene occurs within the epidermal growth factor—like repeat domains in the N terminal of the receptor. Brains of CADASIL patients manifest an aberrant oligomerization of mutant Notch 3 proteins, leading to altered protein–protein interactions [165]. The pathophysiology of CADASIL still remains unknown, but the NF-κB pathway has been reported to play an essential role in the inflammatory responses in the CADASIL-associated angiopathy. NF-κB promotes the expression of genes coding for cytokines that leads to an amplified vascular inflammation level and hence vascular dysfunction [149]. Notch 3 misfolding phenomenon can cause an increase in free radical production in the brain, although the levels produced may not be directly pathogenic [39].

There are also overlapping genes with AD which are known to be involved in the VCI pathogenesis, namely the presenilins, the amyloid precursor protein (APP), and the apolipoprotein E (APOE) [158, 220]. It has been reported that the presence of even a single allele of the *APOE4* variant could be a potential risk factor for progression of VCI [220], thus providing evidence for a commensal interaction between AD and other CVD conditions.

## Unravelling the potential of early detection and intervention strategies

Currently, the treatment options for VCI remain sparse. Understanding the underlying pathophysiology of VCI through CCH provides critical insights to the discovery of biomarkers and targets for disease-modifying treatments.

The identification of specific biomarkers for VCI will be critical for more specific and sensitive diagnosis. These biomarkers may allow for early detection of VCI in at-risk patients. While the diagnostic criteria for VCI is based primarily on neuroimaging, blood-based biomarkers are nevertheless useful as surrogate disease indicators. Many blood and cerebrospinal fluid biomarkers have been identified over the years. Several proinflammatory molecule, such as C-reactive protein, IL-1α and IL-6, have been proposed as potential biomarkers [57, 133, 288]. Given that the increase in plasma level of inflammatory proteins precedes cognitive impairment in VCI, the identification of proinflammatory proteins in early stages of the disease not only offers prognostic advantage but also possible therapeutic intervention [77]. Other than inflammation, the classic marker for blood–brain barrier dysfunction, matrix metalloproteinases, has also been reported multiple times in VCI patients and found to be an early biomarker for cognitive dysfunction [73, 78, 191]. While evidence for oxidative stress in VCI patients are limited, it has been shown that oxidative stress is increased in mild cognitive impairment and AD [44, 210]. Our team has contributed to the field in establishing several possible blood biomarkers for white matter hyperintensities and microinfarcts in clinical cohorts of VCI patients such as serum hepatocyte growth factors, IL-8 and growth differentiation factor-15 [45, 106, 122, 314, 315].

Beyond specific aspects of pathophysiology, several multi-purpose therapeutic interventions have been proposed. The basis of these therapeutic interventions is built upon the evidence that cerebrovascular injury is not always progressive but may instead be reversible. For instance, white matter hyperintensities which are indicative of white matter lesions may regress and be amendable to treatments [87]. Therefore, to the extent that cerebrovascular disease such as white matter hyperintensities is related to CCH and can contribute to the risk of VCI development, markers which allow for the early identification of these lesions may enable early mitigation of cerebrovascular disease and in turn, VCI development. In a similar vein, a deep understanding of the molecular underpinnings of CCH which are relevant to cerebrovascular disease allows for the identification of potential treatment markers as well as drug targets. In the latter case, this then facilitates a potential for development of disease-modifying treatments.

Given that chronic diseases such as VCI are linked to diet and lifestyle factors, interventions at this stage are important for managing the disease. Recent studies have found association of VCI with dietary habits, shedding light on using intermittent fasting as a possible treatment for VCI [201, 280]. Intermittent fasting has been shown to improve cognitive ability, neurotropic factor production, synaptic plasticity, mitochondrial biogenesis, and has also been shown to ameliorate vascular pathology and cognitive impairment in rodent VCI models [11, 85, 110, 213, 237, 273]. Additional systemic beneficial effects of intermittent fasting include attenuation of inflammation, oxidative stress, mitochondrial dysfunction and DNA damage [65]. Separately, in clinical studies on VaD, increasing physical activity has been suggested to reduce the risk of dementia manifestation, albeit not with entirely consistent results due to lack of standardized methods [1, 71, 249, 285].

These approaches are not only potentially disease-modifying at the pathophysiological level, but they also serve as a preventive strategy to mitigate an at-risk patient’s risk of disease. More studies are required for a more robust conclusion in order to delineate the role of intermittent fasting and exercise in VCI clearly [124, 156, 157, 235]. Notably, our team have been investigating the effect of multiple lifestyle interventions on the prevention of cognitive decline under the Singapore Geriatric Intervention Study to Reduce Cognitive Decline and Physical Frailty (SINGER) [52], which is an adaptation of the pioneering Finnish Geriatric Intervention Study to Prevent Cognitive Impairment and Disability (FINGER) [151]. Lifestyle interventions provide a promising potential in managing VCI and reframing the public health perspective of the disease.

## Summary and future directions

VCI is now a widely accepted term introduced to embody the entire spectrum of vascular-related cognitive alterations or cerebrovascular disease-related burdens that can manifest into cognitive impairments [100]. Cognitive deficits associated with VCI include slower mental processing and impaired executive functioning such as poor planning, poor judgement and poor decision-making. Non-cognitive behavioural manifestations include apathy, anxiety and even depression are also common. VCI has been gaining interest in the field as it is potentially preventable, prior to reaching the end-stage dementia [132, 294]. Given such emphasis on early detection and diagnosis in the field, there is a need to better understand the pathophysiology of VCI. As reviewed above, current experimental evidence indicates that a chronic state of hypoperfusion in the brain drives the various pathophysiological mechanisms and structural changes in the brain. CCH therefore holds promise in shedding some light on the molecular and mechanistic underpinnings of VCI.

As reviewed above, there are several common mechanisms that occur during the progression of CCH-induced injury such as energy imbalance, oxidative stress, endoplasmic reticulum stress, mitochondrial dysfunction and inflammation (Fig. 2). These mechanisms drive the downstream structural neuropathological changes in the brain including glial activation, cell death activation, blood–brain barrier breakdown and white matter lesion formation. The pathological features begin from a hypoperfused state and can coexist and interact to adversely influence cognitive function as reported in animal models [25, 27, 43, 166]. This suggests that the effects of CCH on cognition are mediated by mechanistic drivers and structural changes in the brain. Indeed, CCH may be the earliest, insidious indicator of VCI, while brain atrophy and white matter lesions may occur downstream from CCH as more dynamic and detectable changes.

It is exciting to witness the field of VCI rapidly expanding and moving towards sharper definitions and deeper insights into underlying mechanisms. The heterogeneity of the disease is widely recognized to be due to the complex interactions between vascular injuries and risk factors that are involved prior to, and during disease manifestation. Across these subtypes, variations also exist at the clinical, neuroimaging, and pathological levels. Yet, a strong argument may be made that all subtypes of VCI include a CCH state, which we believe to be the main driver for subsequent pathological progression. Prolonged cerebral hypoperfusion may therefore serve as the transition from the at-risk state to the VCI state. The observational and experimental evidence from CCH models presented in this review help reinforce the importance of CCH as a critical feature in our efforts to unravel the underlying molecular mechanisms of VCI. Further identification of specific biomarkers of CCH may provide the rationale for the evaluation of these markers in the clinic which can bring us closer to detecting VCI at an early stage as well as introduce treatment options which may delay disease onset or slow disease progression.

## References

[1] Aarsland D, Saeedzadeh-Sardahaee F, Anderssen S, Ballard C (2010). Is physical activity a potential preventive factor for vascular dementia? A systematic review. Aging Ment Health.

[2] Abbruscato T, Davis T (1999). Protein expression of brain endothelial cell E-cadherin after hypoxia/aglycemia: influence of astrocyte contact. Brain Res.

[3] Abraham JM, Cho L (2010). The homocysteine hypothesis: still relevant to the prevention and treatment of cardiovascular disease?. Cleve Clin J Med.

[4] Aevarsson Ó, Svanborg A, Skoog I (1998). Seven-year survival rate after age 85 years: relation to Alzheimer disease and vascular dementia. Arch Neurol.

[5] Agüero-Torres H, Fratiglioni L, Guo Z, Viitanen M, Winblad B (1999). Mortality from dementia in advanced age: a 5-year follow-up study of incident dementia cases. J Clin Epidemiol.

[6] András IE, Deli MA, Veszelka S, Hayashi K, Hennig B, Toborek M (2007). The NMDA and AMPA/KA receptors are involved in glutamate-induced alterations of occludin expression and phosphorylation in brain endothelial cells. J Cereb Blood Flow Metab.

[7] Anjum I, Fayyaz M, Wajid A, Sohail W, Ali A (2018). Does obesity increase the risk of dementia: a literature review. Cureus.

[8] Anstey KJ, von Sanden C, Salim A, O'Kearney R (2007). Smoking as a risk factor for dementia and cognitive decline: a meta-analysis of prospective studies. Am J Epidemiol.

[9] Appleton JP, Scutt P, Sprigg N, Bath PM (2017). Hypercholesterolaemia and vascular dementia. Clin Sci.

[10] Arai K, Lo EH (2010). Astrocytes protect oligodendrocyte precursor cells via MEK/ERK and PI3K/Akt signaling. J Neurosci Res.

[11] Arguin H, Dionne IJ, Sénéchal M, Bouchard DR, Carpentier AC, Ardilouze J-L, Tremblay A, Leblanc C, Brochu M (2012). Short- and long-term effects of continuous versus intermittent restrictive diet approaches on body composition and the metabolic profile in overweight and obese postmenopausal women: a pilot study. Menopause.

[12] Arnoldussen IA, Kiliaan AJ, Gustafson DR (2014). Obesity and dementia: adipokines interact with the brain. Eur Neuropsychopharmacol.

[13] Aronson MK, Ooi WL, Morgenstern H, Hafner A, Masur D, Crystal H, Frishman WH, Fisher D, Katzman R (1990). Women, myocardial infarction, and dementia in the very old. Neurology.

[14] Arundine M, Tymianski M (2003). Molecular mechanisms of calcium-dependent neurodegeneration in excitotoxicity. Cell Calcium.

[15] Babcock AA, Kuziel WA, Rivest S, Owens T (2003). Chemokine expression by glial cells directs leukocytes to sites of axonal injury in the CNS. J Neurosci.

[16] Baik SH, Selvaraji S, Fann DY, Poh L, Jo DG, Herr DR, Zhang SR, Kim HA, Silva M, Lai MKP (2021). Hippocampal transcriptome profiling reveals common disease pathways in chronic hypoperfusion and aging. Aging (Albany NY).

[17] Bakhru A, Erlinger TP (2005). Smoking cessation and cardiovascular disease risk factors: results from the Third National Health and Nutrition Examination Survey. PLoS Med.

[18] Bakker SLM, de Leeuw FE, de Groot JC, Hofman A, Koudstaal PJ, Breteler MMB (1999). Cerebral vasomotor reactivity and cerebral white matter lesions in the elderly. Neurology.

[19] Barić D (2014). Why pulsatility still matters: a review of current knowledge. Croat Med J.

[20] Barna BP, Pettay J, Barnett GH, Zhou P, Iwasaki K, Estes ML (1994). Regulation of monocyte chemoattractant protein-1 expression in adult human non-neoplastic astrocytes is sensitive to tumor necrosis factor (TNF) or antibody to the 55-kDa TNF receptor. J Neuroimmunol.

[21] Barnes DE, Yaffe K, Byers AL, McCormick M, Schaefer C, Whitmer RA (2012). Midlife vs late-life depressive symptoms and risk of dementia: differential effects for Alzheimer disease and vascular dementia. Arch Gen Psychiatry.

[22] Barreras A, Gurk-Turner C (2003) Angiotensin II receptor blockers. In: Baylor University Medical Center Proceedings, vol 16, pp 123–126. 10.1080/08998280.2003.11927893 10.1080/08998280.2003.11927893 PMC1200815 16278727

[23] Barreto G, White RE, Ouyang Y, Xu L, Giffard RG (2011). Astrocytes: targets for neuroprotection in stroke. Cent Nerv Syst Agents Med Chem.

[24] Belkhelfa M, Beder N, Mouhoub D, Amri M, Hayet R, Nabila T, Bakhti S, Laimouche S, Azzouz D, Belhadj R (2018). The involvement of neuroinflammation and necroptosis in the hippocampus during vascular dementia. J Neuroimmunol.

[25] Ben Ari H, Lifschytz T, Wolf G, Rigbi A, Blumenfeld-Katzir T, Kreisel Merzel T, Koroukhov N, Lotan A, Lerer B (2019). White matter lesions, cerebral inflammation and cognitive function in a mouse model of cerebral hypoperfusion. Brain Res.

[26] Bennett S, Grant MM, Aldred S (2009). Oxidative stress in vascular dementia and Alzheimer's disease: a common pathology. J Alzheimers Dis.

[27] Bennett SA, Tenniswood M, Chen JH, Davidson CM, Keyes MT, Fortin T, Pappas BA (1998). Chronic cerebral hypoperfusion elicits neuronal apoptosis and behavioral impairment. NeuroReport.

[28] Bonifati DM, Kishore U (2007). Role of complement in neurodegeneration and neuroinflammation. Mol Immunol.

[29] Bowling AC, Mutisya EM, Walker LC, Price DL, Cork LC, Beal MH (1993). Age-dependent impairment of mitochondrial function in primate brain. J Neurochem.

[30] Breteler MM, Claus JJ, Grobbee DE, Hofman A (1994). Cardiovascular disease and distribution of cognitive function in elderly people: the Rotterdam study. BMJ.

[31] Brown MD, Wallace DC (1994). Molecular basis of mitochondrial DNA disease. J Bioenerg Biomembr.

[32] Brown RC, Davis TP (2002). Calcium modulation of adherens and tight junction function. Stroke.

[33] Brown WR, Moody DM, Thore CR, Challa VR (2000). Apoptosis in leukoaraiosis. Am J Neuroradiol.

[34] Brown WR, Thore CR (2011). Review: cerebral microvascular pathology in ageing and neurodegeneration. Neuropathol Appl Neurobiol.

[35] Brun A (1994). Pathology and pathophysiology of cerebrovascular dementia: pure subgroups of obstructive and hypoperfusive etiology. Dement Geriatr Cogn Disord.

[36] Bunch TJ, Weiss JP, Crandall BG, May HT, Bair TL, Osborn JS, Anderson JL, Muhlestein JB, Horne BD, Lappe DL (2010). Atrial fibrillation is independently associated with senile, vascular, and Alzheimer's dementia. Heart Rhythm.

[37] Byers AL, Yaffe K (2011). Depression and risk of developing dementia. Nat Rev Neurol.

[38] Camacho A, Massieu L (2006). Role of glutamate transporters in the clearance and release of glutamate during Ischemia and its relation to neuronal death. Arch Med Res.

[39] Campolo J, De Maria R, Mariotti C, Tomasello C, Parolini M, Frontali M, Inzitari D, Valenti R, Federico A, Taroni F (2013). Is the oxidant/antioxidant status altered in CADASIL patients?. PLoS ONE.

[40] Candelario-Jalil E, Thompson J, Taheri S, Grossetete M, Adair JC, Edmonds E, Prestopnik J, Wills J, Rosenberg GA (2011). Matrix metalloproteinases are associated with increased blood–brain barrier opening in vascular cognitive impairment. Stroke.

[41] Castellazzi M, Patergnani S, Donadio M, Giorgi C, Bonora M, Bosi C, Brombo G, Pugliatti M, Seripa D, Zuliani G (2019). Autophagy and mitophagy biomarkers are reduced in sera of patients with Alzheimer’s disease and mild cognitive impairment. Sci Rep.

[42] Catindig J-AS, Venketasubramanian N, Ikram MK, Chen C (2012). Epidemiology of dementia in Asia: insights on prevalence, trends and novel risk factors. J Neurol Sci.

[43] Cechetti F, Pagnussat AS, Worm PV, Elsner VR, Ben J, da Costa MS, Mestriner R, Weis SN, Netto CA (2012). Chronic brain hypoperfusion causes early glial activation and neuronal death, and subsequent long-term memory impairment. Brain Res Bull.

[44] Cervellati C, Romani A, Seripa D, Cremonini E, Bosi C, Magon S, Bergamini CM, Valacchi G, Pilotto A, Zuliani G (2014). Systemic oxidative stress and conversion to dementia of elderly patients with mild cognitive impairment. Biomed Res Int.

[45] Chai YL, Hilal S, Chong JR, Ng YX, Liew OW, Xu X, Ikram MK, Venketasubramanian N, Richards AM, Lai MKP (2016). Growth differentiation factor-15 and white matter hyperintensities in cognitive impairment and dementia. Medicine.

[46] Chai YL, Rajeev V, Poh L, Selvaraji S, Hilal S, Chen CP, Jo DG, Koo EH, Arumugam TV, Lai MK (2022). Chronic cerebral hypoperfusion alters the CypA-EMMPRIN-gelatinase pathway: implications for vascular dementia. J Cereb Blood Flow Metab.

[47] Che H, Yan Y, Kang X-H, Guo F, Yan M-L, Liu H-L, Hou X, Liu T, Zong D-K, Sun L-L (2017). MicroRNA-27a promotes inefficient lysosomal clearance in the hippocampi of rats following chronic brain hypoperfusion. Mol Neurobiol.

[48] Chen C, Homma A, Mok VCT, Krishnamoorthy E, Alladi S, Meguro K, Abe K, Dominguez J, Marasigan S, Kandiah N (2016). Alzheimer's disease with cerebrovascular disease: current status in the Asia–Pacific region. J Intern Med.

[49] Chen CPLH (2004). Transcultural expression of subcortical vascular disease. J Neurol Sci.

[50] Chen L, Deng H, Cui H, Fang J, Zuo Z, Deng J, Li Y, Wang X, Zhao L (2017). Inflammatory responses and inflammation-associated diseases in organs. Oncotarget.

[51] Chen Y, Guo Z, Peng X, Xie W, Chen L, Tan Z (2018). Nimodipine represses AMPK phosphorylation and excessive autophagy after chronic cerebral hypoperfusion in rats. Brain Res Bull.

[52] Chew KA, Xu X, Siongco P, Villaraza S, Phua AKS, Wong ZX, Chung CY, Tang N, Chew E, Henry CJ (2021). SINgapore GERiatric intervention study to reduce physical frailty and cognitive decline (SINGER)-pilot: a feasibility study. Alzheimers Dement (NY).

[53] Choi DH, Lee KH, Kim JH, Seo JH, Kim HY, Shin CY, Han JS, Han SH, Kim YS, Lee J (2014). NADPH oxidase 1, a novel molecular source of ROS in hippocampal neuronal death in vascular dementia. Antioxid Redox Signal.

[54] Choi JC (2015). Genetics of cerebral small vessel disease. J Stroke.

[55] Chrissobolis S, Banfi B, Sobey CG, Faraci FM (2012). Role of Nox isoforms in angiotensin II-induced oxidative stress and endothelial dysfunction in brain. J Appl Physiol (1985).

[56] Ciacciarelli A, Sette G, Giubilei F, Orzi F (2020). Chronic cerebral hypoperfusion: an undefined, relevant entity. J Clin Neurosci.

[57] Cipollini V, Troili F, Giubilei F (2019). Emerging biomarkers in vascular cognitive impairment and dementia: from pathophysiological pathways to clinical application. Int J Mol Sci.

[58] Colcombe S, Kramer AF (2003). Fitness effects on the cognitive function of older adults: a meta-analytic study. Psychol Sci.

[59] Collard CD, Park KA, Montalto MC, Alapati S, Buras JA, Stahl GL, Colgan SP (2002). Neutrophil-derived Glutamate regulates vascular endothelial barrier function. J Biol Chem.

[60] Corrada MM, Brookmeyer R, Berlau D, Paganini-Hill A, Kawas CH (2008). Prevalence of dementia after age 90: results from the 90+ study. Neurology.

[61] Corraini P, Henderson VW, Ording AG, Pedersen L, Horváth-Puhó E, Sørensen HT (2017). Long-term risk of dementia among survivors of ischemic or hemorrhagic stroke. Stroke.

[62] Courties G, Moskowitz MA, Nahrendorf M (2014). The innate immune system after ischemic injury: lessons to be learned from the heart and brain. JAMA Neurol.

[63] Daneman R, Prat A (2015). The blood–brain barrier. Cold Spring Harb Perspect Biol.

[64] Datta A, Qian J, Chong R, Kalaria RN, Francis P, Lai MKP, Chen CP, Sze SK (2014). Novel pathophysiological markers are revealed by iTRAQ-based quantitative clinical proteomics approach in vascular dementia. J Proteom.

[65] de Cabo R, Mattson MP (2019). Effects of intermittent fasting on health, aging, and disease. N Engl J Med.

[66] de la Torre JC (2012). Cardiovascular risk factors promote brain hypoperfusion leading to cognitive decline and dementia. Cardiovasc Psychiatry Neurol.

[67] De Silva TM, Faraci FM (2016). Microvascular dysfunction and cognitive impairment. Cell Mol Neurobiol.

[68] De Vivo DC (1993). The expanding clinical spectrum of mitochondrial diseases. Brain Dev.

[69] Del Ser T, Hachinski V, Merskey H, Munoz DG (1999). An autopsy-verified study of the effect of education on degenerative dementia. Brain.

[70] Ding Q, Dimayuga E, Keller JN (2006). Proteasome regulation of oxidative stress in aging and age-related diseases of the CNS. Antioxid Redox Signal.

[71] Dong J, Zhao J, Lin Y, Liang H, He X, Zheng X, Sui M, Zhuang Z, Yan T (2018). Exercise improves recognition memory and synaptic plasticity in the prefrontal cortex for rats modelling vascular dementia. Neurol Res.

[72] Du J, Ma M, Zhao Q, Fang L, Chang J, Wang Y, Fei R, Song X (2012). Mitochondrial bioenergetic deficits in the hippocampus of rats with chronic Ischemia-induced vascular dementia. Neuroscience.

[73] Duits FH, Hernandez-Guillamon M, Montaner J, Goos JDC, Montañola A, Wattjes MP, Barkhof F, Scheltens P, Teunissen CE, van der Flier WM (2015). Matrix metalloproteinases in Alzheimer’s disease and concurrent cerebral microbleeds. J Alzheimer’s Dis.

[74] Duncombe J, Kitamura A, Hase Y, Ihara M, Kalaria Raj N, Horsburgh K (2017). Chronic cerebral hypoperfusion: a key mechanism leading to vascular cognitive impairment and dementia. Closing the translational gap between rodent models and human vascular cognitive impairment and dementia. Clin Sci.

[75] Duron E, Hanon O (2008). Vascular risk factors, cognitive decline, and dementia. Vasc Health Risk Manag.

[76] Enciu A-M, Constantinescu SN, Popescu LM, Mureşanu DF, Popescu BO (2011). Neurobiology of vascular dementia. J Aging Res.

[77] Engelhart MJ, Geerlings MI, Meijer J, Kiliaan A, Ruitenberg A, van Swieten JC, Stijnen T, Hofman A, Witteman JCM, Breteler MMB (2004). Inflammatory proteins in plasma and the risk of dementia: the rotterdam study. Arch Neurol.

[78] Erhardt EB, Pesko JC, Prestopnik J, Thompson J, Caprihan A, Rosenberg GA (2018). Biomarkers identify the Binswanger type of vascular cognitive impairment. J Cereb Blood Flow Metab.

[79] Esposito M, Sherr GL (2019). Epigenetic modifications in Alzheimer's neuropathology and therapeutics. Front Neurosci.

[80] Famulari AL, Marschoff ER, Llesuy SF, Kohan S, Serra JA, Domínguez RO, Repetto MG, Reides CG, de Lustig ES (1996). The antioxidant enzymatic blood profile in Alzheimer's and vascular diseases. Their association and a possible assay to differentiate demented subjects and controls. J Neurol Sci.

[81] Fan Y, Ou X, Wang W, Tian X, Yan S, Hu N, Zhang X, Xing W (2017). Identification and analysis of toll like receptor 4 (TLR4) level changes in vascular dementia patients related type 2 diabetes mellitus. Biomed Res.

[82] Fann DY-W, Lee S-Y, Manzanero S, Chunduri P, Sobey CG, Arumugam TV (2013). Pathogenesis of acute stroke and the role of inflammasomes. Ageing Res Rev.

[83] Faraci F (2006). Reactive oxygen species: influence on cerebral vascular tone. J Appl Physiol.

[84] Favaloro B, Allocati N, Graziano V, Di Ilio C, De Laurenzi V (2012). Role of apoptosis in disease. Aging.

[85] Fernando HA, Zibellini J, Harris RA, Seimon RV, Sainsbury A (2019). Effect of Ramadan fasting on weight and body composition in healthy non-athlete adults: a systematic review and meta-analysis. Nutrients.

[86] Fernando MS, Simpson JE, Matthews F, Brayne C, Lewis CE, Barber R, Kalaria RN, Forster G, Esteves F, Wharton SB (2006). White matter lesions in an unselected cohort of the elderly. Stroke.

[87] Filley CM (2021). Cognitive dysfunction in white matter disorders: new perspectives in treatment and recovery. J Neuropsychiatry Clin Neurosci.

[88] Fischer S, Wobben M, Kleinstück J, Renz D, Schaper W (2000). Effect of astroglial cells on hypoxia-induced permeability in PBMEC cells. Am J Physiol Cell Physiol.

[89] Förstermann U (2010). Nitric oxide and oxidative stress in vascular disease. Pflüg Arch Eur J Physiol.

[90] Freiheit EA, Hogan DB, Eliasziw M, Patten SB, Demchuk AM, Faris P, Anderson T, Galbraith D, Parboosingh JS, Ghali WA (2012). A dynamic view of depressive symptoms and neurocognitive change among patients with coronary artery disease. Arch Gen Psychiatry.

[91] French HM, Reid M, Mamontov P, Simmons RA, Grinspan JB (2009). Oxidative stress disrupts oligodendrocyte maturation. J Neurosci Res.

[92] Friedman L (2007). CALCIUM: a role for neuroprotection and sustained adaptation. Mol Interv.

[93] Fünfschilling U, Supplie L, Mahad D, Boretius S, Saab A, Edgar J, Brinkmann B, Kassmann C, Tzvetanova I, Möbius W (2012). Glycolytic oligodendrocytes maintain myelin and long-term axonal integrity. Nature.

[94] Gannon OJ, Robison LS, Custozzo AJ, Zuloaga KL (2019). Sex differences in risk factors for vascular contributions to cognitive impairment & dementia. Neurochem Int.

[95] Gemma C, Vila J, Bachstetter A, Bickford PC (2007). Oxidative stress and the aging brain: from theory to prevention.

[96] González-Mariscal L, Tapia R, Chamorro D (2008). Crosstalk of tight junction components with signaling pathways. Biochim Biophys Acta.

[97] Gordon P, Flanagan P (2016). Smoking: a risk factor for vascular disease. J Vasc Nurs.

[98] Gorelick PB (2004). Risk factors for vascular dementia and Alzheimer disease. Stroke.

[99] Gorelick PB, Counts SE, Nyenhuis D (2016). Vascular cognitive impairment and dementia. Biochim Biophys Acta (BBA) Mol Basis Dis.

[100] Gorelick PB, Scuteri A, Black SE, Decarli C, Greenberg SM, Iadecola C, Launer LJ, Laurent S, Lopez OL, Nyenhuis D (2011). Vascular contributions to cognitive impairment and dementia: a statement for healthcare professionals from the American Heart Association/American Stroke Association. Stroke.

[101] Graban A, Bednarska-Makaruk M, Bochyńska A, Lipczyńska-Łojkowska W, Ryglewicz D, Wehr H (2009). Vascular and biochemical risk factors of vascular dementia after lacunar strokes (S-VaD) and after multiinfarcts in strategic areas (M-VaD). J Neurol Sci.

[102] Graeber MB, Grasbon-Frodl E, Eitzen UV, Kösel S (1998). Neurodegeneration and aging: role of the second genome. J Neurosci Res.

[103] Gray F, Polivka M, Viswanathan A, Baudrimont M, Bousser M-G, Chabriat H (2007). Apoptosis in cerebral autosomal-dominant arteriopathy with subcortical infarcts and leukoencephalopathy. J Neuropathol Exp Neurol.

[104] Grimm A, Friedland K, Eckert A (2016). Mitochondrial dysfunction: the missing link between aging and sporadic Alzheimer's disease. Biogerontology.

[105] Grivennikov S, Karin M (2010). Dangerous liaisons: STAT3 and NF-kappaB collaboration and crosstalk in cancer. Cytokine Growth Factor Rev.

[106] Gyanwali B, Lai MKP, Lui B, Liew OW, Venketasubramanian N, Richards AM, Chen C, Hilal S (2021). Blood-based cardiac biomarkers and the risk of cognitive decline, cerebrovascular disease, and clinical events. Stroke.

[107] Gyanwali B, Shaik MA, Tan BY, Venketasubramanian N, Chen C, Hilal S (2019). Risk factors for and clinical relevance of incident and progression of cerebral small vessel disease markers in an Asian memory clinic population. J Alzheimer's Dis.

[108] Hachinski V, Iadecola C, Petersen RC, Breteler MM, Nyenhuis DL, Black SE, Powers WJ, DeCarli C, Merino JG, Kalaria RN (2006). National Institute of Neurological Disorders and Stroke-Canadian Stroke Network vascular cognitive impairment harmonization standards. Stroke.

[109] Hainsworth AH, Yeo NE, Weekman EM, Wilcock DM (2016). Homocysteine, hyperhomocysteinemia and vascular contributions to cognitive impairment and dementia (VCID). Biochim Biophys Acta.

[110] Halberg N, Henriksen M, Söderhamn N, Stallknecht B, Ploug T, Schjerling P, Dela F (2005). Effect of intermittent fasting and refeeding on insulin action in healthy men. J Appl Physiol.

[111] Halling A, Berglund J (2006). Association of diagnosis of ischaemic heart disease, diabetes mellitus and heart failure with cognitive function in the elderly population. Eur J Gen Pract.

[112] Haorah J, Ramirez SH, Schall K, Smith D, Pandya R, Persidsky Y (2007). Oxidative stress activates protein tyrosine kinase and matrix metalloproteinases leading to blood–brain barrier dysfunction. J Neurochem.

[113] Harter K, Levine M, Henderson SO (2015). Anticoagulation drug therapy: a review. West J Emerg Med.

[114] Hattori Y, Enmi J, Kitamura A, Yamamoto Y, Saito S, Takahashi Y, Iguchi S, Tsuji M, Yamahara K, Nagatsuka K (2015). A novel mouse model of subcortical infarcts with dementia. J Neurosci.

[115] Hayashi M, Luo Y, Laning J, Strieter RM, Dorf ME (1995). Production and function of monocyte chemoattractant protein-1 and other β-chemokines in murine glial cells. J Neuroimmunol.

[116] Helmer C, Joly P, Letenneur L, Commenges D, Dartigues JF (2001). Mortality with dementia: results from a French prospective community-based cohort. Am J Epidemiol.

[117] Hénon H, Durieu I, Guerouaou D, Lebert F, Pasquier F, Leys D (2001). Poststroke dementia: incidence and relationship to prestroke cognitive decline. Neurology.

[118] Hertz L (2008). Bioenergetics of cerebral ischemia: a cellular perspective. Neuropharmacology.

[119] Hetz C, Dienel GA (2002). Energy metabolism in the brain.

[120] Hetz C, Saxena S (2017). ER stress and the unfolded protein response in neurodegeneration. Nat Rev Neurol.

[121] High FA, Epstein JA (2008). The multifaceted role of Notch in cardiac development and disease. Nat Rev Genet.

[122] Hilal S, Chai YL, van Veluw S, Shaik MA, Ikram MK, Venketasubramanian N, Richards AM, Biessels GJ, Chen C (2017). Association between subclinical cardiac biomarkers and clinically manifest cardiac diseases with cortical cerebral microinfarcts. JAMA Neurol.

[123] Hilal S, Mok V, Youn YC, Wong A, Ikram MK, Chen CLH (2017). Prevalence, risk factors and consequences of cerebral small vessel diseases: data from three Asian countries. J Neurol Neurosurg Psychiatry.

[124] Ho SC, Woo J, Sham A, Chan SG, Yu ALM (2001). A 3-year follow-up study of social, lifestyle and health predictors of cognitive impairment in a Chinese older cohort. Int J Epidemiol.

[125] Hsu M-J, Sheu J-R, Lin C-H, Shen M-Y, Hsu CY (2010). Mitochondrial mechanisms in amyloid beta peptide-induced cerebrovascular degeneration. Biochim Biophys Acta (BBA) Gen Subj.

[126] Hu M, Liu Z, Lv P, Wang H, Zhu Y, Qi Q, Xu J (2017). Autophagy and Akt/CREB signalling play an important role in the neuroprotective effect of nimodipine in a rat model of vascular dementia. Behav Brain Res.

[127] Huang J, Li J, Feng C, Huang X, Wong L, Liu X, Nie Z, Xi G (2018). Blood–brain barrier damage as the starting point of leukoaraiosis caused by cerebral chronic hypoperfusion and its involved mechanisms: effect of agrin and aquaporin-4. Biomed Res Int.

[128] Huang J, Zhang Z, Hong X, Wang J, Wei J, Wen H (2009). Early cognitive predictors of vascular dementia: a population-based longitudinal study in Chinese elderly. J Exp Stroke Transl Med.

[129] Hundal RS, Krssak M, Dufour S, Laurent D, Lebon V, Chandramouli V, Inzucchi SE, Schumann WC, Petersen KF, Landau BR (2000). Mechanism by which metformin reduces glucose production in type 2 diabetes. Diabetes.

[130] Iadecola C (2013). The pathobiology of vascular dementia. Neuron.

[131] Ikram MA, Bersano A, Manso-Calderón R, Jia JP, Schmidt H, Middleton L, Nacmias B, Siddiqi S, Adams HH (2017). Genetics of vascular dementia—review from the ICVD working group. BMC Med.

[132] Ingles JL, Wentzel C, Fisk JD, Rockwood K (2002). Neuropsychological predictors of incident dementia in patients with vascular cognitive impairment, without dementia. Stroke.

[133] Jagtap A, Gawande S, Sharma S (2015). Biomarkers in vascular dementia: a recent update. Biomark Genom Med.

[134] Jiang XL, Samant S, Lesko LJ, Schmidt S (2015). Clinical pharmacokinetics and pharmacodynamics of clopidogrel. Clin Pharmacokinet.

[135] Jørgensen IF, Aguayo-Orozco A, Lademann M, Brunak S (2020). Age-stratified longitudinal study of Alzheimer's and vascular dementia patients. Alzheimers Dement.

[136] Juma W, Lira A, Marzuk A, Marzuk Z, Hakim A, Thompson C (2011). C-reactive protein expression in a rodent model of chronic cerebral hypoperfusion. Brain Res.

[137] Justin BN, Turek M, Hakim AM (2013). Heart disease as a risk factor for dementia. Clin Epidemiol.

[138] Kakae M, Tobori S, Morishima M, Nagayasu K, Shirakawa H, Kaneko S (2019). Depletion of microglia ameliorates white matter injury and cognitive impairment in a mouse chronic cerebral hypoperfusion model. Biochem Biophys Res Commun.

[139] Kalaria RN (2016). Neuropathological diagnosis of vascular cognitive impairment and vascular dementia with implications for Alzheimer's disease. Acta Neuropathol.

[140] Kalaria RN (2018). The pathology and pathophysiology of vascular dementia. Neuropharmacology.

[141] Kalogeris T, Baines CP, Krenz M, Korthuis RJ (2012). Cell biology of ischemia/reperfusion injury. Int Rev Cell Mol Biol.

[142] Kang JH, Cook N, Manson J, Buring JE, Grodstein F (2007). Low dose aspirin and cognitive function in the women’s health study cognitive cohort. BMJ.

[143] Katsura K-I, Kristián T, Smith M-L, Siesjö BK (1994). Acidosis induced by hypercapnia exaggerates ischemic brain damage. J Cereb Blood Flow Metab.

[144] Kawahara M, Sadakane Y, Koyama H, Konoha K, Ohkawara S (2013). D-histidine and L-histidine attenuate zinc-induced neuronal death in GT1–7 cells. Metallomics Integr Biomet Sci.

[145] Kawamura J, Meyer JS, Terayama Y, Weathers S (1991). Leukoaraiosis correlates with cerebral hypoperfusion in vascular dementia. Stroke.

[146] Kawasaki T, Kawai T (2014). Toll-like receptor signaling pathways. Front Immunol.

[147] Khan A, Kalaria RN, Corbett A, Ballard C (2016). Update on vascular dementia. J Geriatr Psychiatry Neurol.

[148] Kiliaan AJ, Arnoldussen IA, Gustafson DR (2014). Adipokines: a link between obesity and dementia?. Lancet Neurol.

[149] Killeen MJ, Linder M, Pontoniere P, Crea R (2014). NF-κβ signaling and chronic inflammatory diseases: exploring the potential of natural products to drive new therapeutic opportunities. Drug Discovery Today.

[150] Kim S, Choi SH, Lee YM, Kim MJ, Kim YD, Kim JY, Park JH, Myung W, Na HR, Han HJ (2015). Periventricular white matter hyperintensities and the risk of dementia: a CREDOS study. Int Psychogeriatr.

[151] Kivipelto M, Mangialasche F, Snyder HM, Allegri R, Andrieu S, Arai H, Baker L, Belleville S, Brodaty H, Brucki SM (2020). World-Wide FINGERS Network: a global approach to risk reduction and prevention of dementia. Alzheimers Dement.

[152] Kokko JP (1984). Site and mechanism of action of diuretics. Am J Med.

[153] Kokmen E, Whisnant JP, O'Fallon WM, Chu CP, Beard CM (1996). Dementia after ischemic stroke: a population-based study in Rochester, Minnesota (1960–1984). Neurology.

[154] Kuller LH, Lopez OL, Jagust WJ, Becker JT, DeKosky ST, Lyketsos C, Kawas C, Breitner JC, Fitzpatrick A, Dulberg C (2005). Determinants of vascular dementia in the Cardiovascular Health Cognition Study. Neurology.

[155] Lanza G, Cantone M, Musso S, Borgione E, Scuderi C, Ferri R (2018). Early-onset subcortical ischemic vascular dementia in an adult with mtDNA mutation 3316G>A. J Neurol.

[156] Laurin D, Verreault R, Lindsay J, MacPherson K, Rockwood K (2001). Physical activity and risk of cognitive impairment and dementia in elderly persons. Arch Neurol.

[157] Lautenschlager NT, Cox K, Cyarto EV (2012). The influence of exercise on brain aging and dementia. Biochim Biophys Acta (BBA) Mol Basis Dis.

[158] Leblanc GG, Meschia JF, Stuss DT, Hachinski V (2006). Genetics of vascular cognitive impairment. Stroke.

[159] Lejri I, Grimm A, Eckert A (2018). Mitochondria, estrogen and female brain aging. Front Aging Neurosci.

[160] Lenglet S, Montecucco F, Mach F, Schaller K, Gasche Y, Copin J-C (2017). Analysis of the expression of nine secreted matrix metalloproteinases and their endogenous inhibitors in the brain of mice subjected to ischaemic stroke. Thromb Haemost.

[161] Leuner K, Müller W, Reichert A (2012). From mitochondrial dysfunction to amyloid beta formation: novel insights into the pathogenesis of Alzheimer’s disease. Mol Neurobiol.

[162] Li H, Liu Y, Lin LT, Wang XR, Du SQ, Yan CQ, He T, Yang JW, Liu CZ (2016). Acupuncture reversed hippocampal mitochondrial dysfunction in vascular dementia rats. Neurochem Int.

[163] Lin J, Wang D, Lan L, Fan Y (2017). Multiple factors involved in the pathogenesis of white matter lesions. Biomed Res Int.

[164] Lindén T, Skoog I, Fagerberg B, Steen B, Blomstrand C (2004). Cognitive impairment and dementia 20 months after stroke. Neuroepidemiology.

[165] Ling C, Liu Z, Song M, Zhang W, Wang S, Liu X, Ma S, Sun S, Fu L, Chu Q (2019). Modeling CADASIL vascular pathologies with patient-derived induced pluripotent stem cells. Protein Cell.

[166] Liu H, Zhang J (2012). Cerebral hypoperfusion and cognitive impairment: the pathogenic role of vascular oxidative stress. Int J Neurosci.

[167] Liu Q, He S, Groysman L, Shaked D, Russin J, Scotton TC, Cen S, Mack WJ (2013). White matter injury due to experimental chronic cerebral hypoperfusion is associated with C5 deposition. PLoS ONE.

[168] Ma Y, Liu Y, Zhang Z, Yang G-Y (2019). Significance of complement system in ischemic stroke: a comprehensive review. Aging Dis.

[169] Magami S, Miyamoto N, Ueno Y, Hira K, Tanaka R, Yamashiro K, Oishi H, Arai H, Urabe T, Hattori N (2019). The effects of astrocyte and oligodendrocyte lineage cell interaction on white matter injury under chronic cerebral hypoperfusion. Neuroscience.

[170] Maloney B, Lahiri DK (2016). Epigenetics of dementia: understanding the disease as a transformation rather than a state. Lancet Neurol.

[171] Man S, Ubogu E, Ransohoff R (2007). Inflammatory cell migration into the central nervous system: a few new twists on an old tale. Brain Pathol.

[172] Mancuso C, Scapagini G, Currò D, Stella AMG, Marco CD, Butterfield DA, Calabrese V (2007). Mitochondrial dysfunction, free radical generation and cellular stress response in neurodegenerative disorders. FBL.

[173] Markus HS, Schmidt R (2019). Genetics of vascular cognitive impairment. Stroke.

[174] Masumura M, Hata R, Nagai Y, Sawada T (2001). Oligodendroglial cell death with DNA fragmentation in the white matter under chronic cerebral hypoperfusion: comparison between normotensive and spontaneously hypertensive rats. Neurosci Res.

[175] Mathey E, Park S, Hughes R, Pollard J, Armati P, Barnett M, Taylor B, Dyck P, Kiernan M, Lin C (2015). Chronic inflammatory demyelinating polyradiculoneuropathy: from pathology to phenotype. J Neurol Neurosurg Psychiatry.

[176] Matsushita K, Kuriyama Y, Nagatsuka K, Nakamura M, Sawada T, Omae T (1994). Periventricular white matter lucency and cerebral blood flow autoregulation in hypertensive patients. Hypertension.

[177] Matsuyama H, Shindo A, Shimada T, Yata K, Wakita H, Takahashi R, Tomimoto H (2020). Chronic cerebral hypoperfusion activates AIM2 and NLRP3 inflammasome. Brain Res.

[178] Matute C (2011). Glutamate and ATP signalling in white matter pathology. J Anat.

[179] Matute C, Ransom BR (2012). Roles of white matter in central nervous system pathophysiologies. ASN Neuro.

[180] McAleese KE, Alafuzoff I, Charidimou A, De Reuck J, Grinberg LT, Hainsworth AH, Hortobagyi T, Ince P, Jellinger K, Gao J (2016). Post-mortem assessment in vascular dementia: advances and aspirations. BMC Med.

[181] McQueen J, Reimer M, Holland P, Manso Y, McLaughlin M, Fowler J, Horsburgh K (2014). Restoration of oligodendrocyte pools in a mouse model of chronic cerebral hypoperfusion. PLoS ONE.

[182] Mekaj YH, Daci FT, Mekaj AY (2015). New insights into the mechanisms of action of aspirin and its use in the prevention and treatment of arterial and venous thromboembolism. Ther Clin Risk Manag.

[183] Miyamoto N, Magami S, Inaba T, Ueno Y, Hira K, Kijima C, Nakajima S, Yamashiro K, Urabe T, Hattori N (2020). The effects of A1/A2 astrocytes on oligodendrocyte linage cells against white matter injury under prolonged cerebral hypoperfusion. Glia.

[184] Miyamoto N, Maki T, Shindo A, Liang AC, Maeda M, Egawa N, Itoh K, Lo EK, Lok J, Ihara M (2015). Astrocytes promote oligodendrogenesis after white matter damage via brain-derived neurotrophic factor. J Neurosci.

[185] Montagne A, Nikolakopoulou AM, Zhao Z, Sagare AP, Si G, Lazic D, Barnes SR, Daianu M, Ramanathan A, Go A (2018). Pericyte degeneration causes white matter dysfunction in the mouse central nervous system. Nat Med.

[186] Morris MC (2016). Nutrition and risk of dementia: overview and methodological issues. Ann N Y Acad Sci.

[187] Morris MC (2012). Nutritional determinants of cognitive aging and dementia. Proc Nutr Soc.

[188] Morris MC, Tangney CC (2014). Dietary fat composition and dementia risk. Neurobiol Aging.

[189] Mracskó E, Hugyecz M, Institoris A, Farkas E, Bari F (2009). Changes in pro-oxidant and antioxidant enzyme levels during cerebral hypoperfusion in rats. Brain Res.

[190] Nagai M, Hoshide S, Kario K (2010). Hypertension and dementia. Am J Hypertens.

[191] Nation DA, Sweeney MD, Montagne A, Sagare AP, D’Orazio LM, Pachicano M, Sepehrband F, Nelson AR, Buennagel DP, Harrington MG (2019). Blood–brain barrier breakdown is an early biomarker of human cognitive dysfunction. Nat Med.

[192] Navarro A, Boveris A (2007). The mitochondrial energy transduction system and the aging process. Am J Physiol Cell Physiol.

[193] Newman AB, Fitzpatrick AL, Lopez O, Jackson S, Lyketsos C, Jagust W, Ives D, Dekosky ST, Kuller LH (2005). Dementia and Alzheimer's disease incidence in relationship to cardiovascular disease in the Cardiovascular Health Study cohort. J Am Geriatr Soc.

[194] Nishio K, Ihara M, Yamasaki N, Kalaria R, Maki T, Fujita Y, Ito H, Oishi N, Fukuyama H, Miyakawa T (2010). A mouse model characterizing features of vascular dementia with hippocampal atrophy. Stroke J Cereb Circ.

[195] Niu XL, Jiang X, Xu GD, Zheng GM, Tang ZP, Yin N, Li XQ, Yang YY, Lv PY (2018). DL-3-n-butylphthalide alleviates vascular cognitive impairment by regulating endoplasmic reticulum stress and the Shh/Ptch1 signaling-pathway in rats. J Cell Physiol.

[196] Nunnari J, Suomalainen A (2012). Mitochondria: in sickness and in health. Cell.

[197] O’Barr S, Cooper NR (2000). The C5a complement activation peptide increases IL-1β and IL-6 release from amyloid-β primed human monocytes: implications for Alzheimer’s disease. J Neuroimmunol.

[198] O’Sullivan M, Lythgoe DJ, Pereira AC, Summers PE, Jarosz JM, Williams SCR, Markus HS (2002). Patterns of cerebral blood flow reduction in patients with ischemic leukoaraiosis. Neurology.

[199] Ogino S, Lochhead P, Chan AT, Nishihara R, Cho E, Wolpin BM, Meyerhardt JA, Meissner A, Schernhammer ES, Fuchs CS (2013). Molecular pathological epidemiology of epigenetics: emerging integrative science to analyze environment, host, and disease. Mod Pathol Off J US Can Acad Pathol Inc.

[200] Ott A, Breteler MM, van Harskamp F, Claus JJ, van der Cammen TJ, Grobbee DE, Hofman A (1995). Prevalence of Alzheimer's disease and vascular dementia: association with education. The Rotterdam study. BMJ.

[201] Ozawa M, Ninomiya T, Ohara T, Doi Y, Uchida K, Shirota T, Yonemoto K, Kitazono T, Kiyohara Y (2013). Dietary patterns and risk of dementia in an elderly Japanese population: the Hisayama Study. Am J Clin Nutr.

[202] Paciaroni M, Bogousslavsky J (2013). Connecting cardiovascular disease and dementia: further evidence. J Am Heart Assoc.

[203] Park J-M, Kim YJ, Song MK, Lee J-M, Kim Y-J (2018). Genome-wide DNA methylation profiling in a rat model with vascular dementia. Mol Med Rep.

[204] Peila R, Rodriguez BL, Launer LJ (2002). Type 2 diabetes, APOE gene, and the risk for dementia and related pathologies: the Honolulu-Asia aging study. Diabetes.

[205] Phillips NA, Mate-Kole CC (1997). Cognitive deficits in peripheral vascular disease. A comparison of mild stroke patients and normal control subjects. Stroke.

[206] Poh L, Fann DY, Wong P, Lim HM, Foo SL, Kang S-W, Rajeev V, Selvaraji S, Iyer VR, Parathy N (2021). AIM2 inflammasome mediates hallmark neuropathological alterations and cognitive impairment in a mouse model of vascular dementia. Mol Psychiatry.

[207] Poh L, Razak S, Lim HM, Lai MKP, Chen CL, Lim LHK, Arumugam TV, Fann DY (2021). AIM2 inflammasome mediates apoptotic and pyroptotic death in the cerebellum following chronic hypoperfusion. Exp Neurol.

[208] Portegies ML, Wolters FJ, Hofman A, Ikram MK, Koudstaal PJ, Ikram MA (2016). Prestroke vascular pathology and the risk of recurrent stroke and poststroke dementia. Stroke.

[209] Posner HB, Tang MX, Luchsinger J, Lantigua R, Stern Y, Mayeux R (2002). The relationship of hypertension in the elderly to AD, vascular dementia, and cognitive function. Neurology.

[210] Praticò D, Clark CM, Liun F, Lee VYM, Trojanowski JQ (2002). Increase of brain oxidative stress in mild cognitive impairment: a possible predictor of Alzheimer disease. Arch Neurol.

[211] Price BR, Wilcock DM, Weekman EM (2018). Hyperhomocysteinemia as a risk factor for vascular contributions to cognitive impairment and dementia. Front Aging Neurosci.

[212] Rafnsson SB, Deary IJ, Fowkes FG (2009). Peripheral arterial disease and cognitive function. Vasc Med.

[213] Rajeev V, Fann DY, Dinh QN, Kim HA, De Silva TM, Jo DG, Drummond GR, Sobey CG, Lai MKP, Chen CL (2022). Intermittent fasting attenuates Hallmark vascular and neuronal pathologies in a mouse model of vascular cognitive impairment. Int J Biol Sci.

[214] Rajeev V, Fann DY, Dinh QN, Kim HA, De Silva TM, Lai MKP, Chen CL, Drummond GR, Sobey CG, Arumugam TV (2022). Pathophysiology of blood brain barrier dysfunction during chronic cerebral hypoperfusion in vascular cognitive impairment. Theranostics.

[215] Ravaglia G, Forti P, Maioli F, Chiappelli M, Montesi F, Tumini E, Mariani E, Licastro F, Patterson C (2007). Blood inflammatory markers and risk of dementia: the conselice study of brain aging. Neurobiol Aging.

[216] Ravona-Springer R, Schnaider-Beeri M (2011). The association of diabetes and dementia and possible implications for nondiabetic populations. Expert Rev Neurother.

[217] Reimer M, McQueen J, Searcy L, Scullion G, Desmazières A, Holland P, Smith J, Gliddon C, Wood E, Herzyk P (2011). Rapid disruption of axon-glial integrity in response to mild cerebral hypoperfusion. J Neurosci Off J Soc Neurosci.

[218] Rizzuto R, De Stefani D, Raffaello A, Mammucari C (2012). Mitochondria as sensors and regulators of calcium signalling. Nat Rev Mol Cell Biol.

[219] Roberts RO, Knopman DS, Geda YE, Cha RH, Roger VL, Petersen RC (2010). Coronary heart disease is associated with non-amnestic mild cognitive impairment. Neurobiol Aging.

[220] Rohn TT, McCarty KL, Love JE, Head E (2014). Is apolipoprotein E4 an important risk factor for dementia in persons with down syndrome?. J Parkinson's Dis Alzheimer's Dis.

[221] Román GC (2003). Stroke, cognitive decline and vascular dementia: the silent epidemic of the 21st century. Neuroepidemiology.

[222] Román GC (2002). Vascular dementia may be the most common form of dementia in the elderly. J Neurol Sci.

[223] Rosenberg GA, Sullivan N, Esiri MM (2001). White matter damage is associated with matrix metalloproteinases in vascular dementia. Stroke.

[224] Ruitenberg A, Den Heijer T, Bakker SLM, Van Swieten JC, Koudstaal PJ, Hofman A, Breteler MMB (2005). Cerebral hypoperfusion and clinical onset of dementia: the Rotterdam study. Ann Neurol.

[225] Russ TC, Stamatakis E, Hamer M, Starr JM, Kivimäki M, Batty GD (2013). Socioeconomic status as a risk factor for dementia death: individual participant meta-analysis of 86508 men and women from the UK. Br J Psychiatry.

[226] Rygiel K (2016). Can angiotensin-converting enzyme inhibitors impact cognitive decline in early stages of Alzheimer's disease? An overview of research evidence in the elderly patient population. J Postgrad Med.

[227] Sabayan B, Jansen S, Oleksik AM, van Osch MJP, van Buchem MA, van Vliet P, de Craen AJM, Westendorp RGJ (2012). Cerebrovascular hemodynamics in Alzheimer's disease and vascular dementia: a meta-analysis of transcranial Doppler studies. Ageing Res Rev.

[228] Sachdev P, Kalaria R, O’Brien J, Skoog I, Alladi S, Black SE, Blacker D, Blazer D, Chen C, Chui H (2014). Diagnostic criteria for vascular cognitive disorders: a VASCOG statement. Alzheimer Dis Assoc Disord.

[229] Saito A (2014). Physiological functions of endoplasmic reticulum stress transducer OASIS in central nervous system. Anat Sci Int.

[230] Saridin FN, Hilal S, Villaraza SG, Reilhac A, Gyanwali B, Tanaka T, Stephenson MC, Ng SL, Vrooman H, van der Flier WM (2020). Brain amyloid β, cerebral small vessel disease, and cognition. Neurology.

[231] Scheel P, Puls I, Becker G, Schöning M (1999). Volume reduction in cerebral blood flow in patients with vascular dementia. Lancet.

[232] Schmidt R, Schmidt H, Curb J, Masaki K, White L, Launer L (2002). Early inflammation and dementia: a 25-year follow-up of the Honolulu-Asia aging study. Ann Neurol.

[233] Schmitz T, Chew L-J (2008). Cytokines and myelination in the central nervous system. TheScientificWorldJOURNAL.

[234] Schuff N, Matsumoto S, Kmiecik J, Studholme C, Du A, Ezekiel F, Miller BL, Kramer JH, Jagust WJ, Chui HC (2009). Cerebral blood flow in ischemic vascular dementia and Alzheimer's disease, measured by arterial spin-labeling magnetic resonance imaging. Alzheimers Dement.

[235] Schuit AJ, Feskens MEJ, Launer LJ, Kromhout D (2001). Physical activity and cognitive decline, the role of the apolipoprotein e4 allele. Med Sci Sports Exerc.

[236] Schwarzinger M, Pollock BG, Hasan OSM, Dufouil C, Rehm J (2018). Contribution of alcohol use disorders to the burden of dementia in France 2008–13: a nationwide retrospective cohort study. Lancet Public Health.

[237] Selvaraji S, Efthymios M, Foo RSY, Fann DY, Lai MKP, Chen CLH, Lim KL, Arumugam TV (2022). Time-restricted feeding modulates the DNA methylation landscape, attenuates hallmark neuropathology and cognitive impairment in a mouse model of vascular dementia. Theranostics.

[238] Shabir O, Berwick J, Francis S (2018). Neurovascular dysfunction in vascular dementia, Alzhiemers and atherosclerosis. BMC Neurosci.

[239] Sharp ES, Gatz M (2011). Relationship between education and dementia: an updated systematic review. Alzheimer Dis Assoc Disord.

[240] Shen Y, Halperin JA, Lee C-M (1995). Complement-mediated neurotoxicity is regulated by homologous restriction. Brain Res.

[241] Shi G-X, Liu C-Z, Wang L-P, Guan L-P, Li S-Q (2012). Biomarkers of oxidative stress in vascular dementia patients. Can J Neurol Sci J Can Sci Neurol.

[242] Shibata M, Ohtani R, Ihara M, Tomimoto H (2004). White matter lesions and glial activation in a novel mouse model of chronic cerebral hypoperfusion. Stroke.

[243] Shibata M, Yamasaki N, Miyakawa T, Kalaria RN, Fujita Y, Ohtani R, Ihara M, Takahashi R, Tomimoto H (2007). Selective impairment of working memory in a mouse model of chronic cerebral hypoperfusion. Stroke.

[244] Silva IVG, de Figueiredo RC, Rios DRA (2019). Effect of different classes of antihypertensive drugs on endothelial function and inflammation. Int J Mol Sci.

[245] Simpson JE, Fernando MS, Clark L, Ince PG, Matthews F, Forster G, O'Brien JT, Barber R, Kalaria RN, Brayne C (2007). White matter lesions in an unselected cohort of the elderly: astrocytic, microglial and oligodendrocyte precursor cell responses. Neuropathol Appl Neurobiol.

[246] Skoog I (1998). Status of risk factors for vascular dementia. Neuroepidemiology.

[247] Skrobot OA, Attems J, Esiri M, Hortobágyi T, Ironside JW, Kalaria RN, King A, Lammie GA, Mann D, Neal J (2016). Vascular cognitive impairment neuropathology guidelines (VCING): the contribution of cerebrovascular pathology to cognitive impairment. Brain.

[248] Sofroniew MV (2014). Astrogliosis. Cold Spring Harb Perspect Biol.

[249] Son H-H, Pt OhMS, Jung L, Pt PMS, Rae J, Pt PhD (2010). The effect of an exercise program on activities of daily living (ADL), balance and cognition in elderly individuals with Alzheimer’s disease and vascular dementia. J Korean Phys Ther.

[250] Stamatovic SM, Johnson AM, Keep RF, Andjelkovic AV (2016). Junctional proteins of the blood–brain barrier: new insights into function and dysfunction. Tissue Barriers.

[251] Stampfer MJ, Willett WC, Colditz GA, Rosner B, Speizer FE, Hennekens CH (1985). A prospective study of postmenopausal estrogen therapy and coronary heart disease. N Engl J Med.

[252] Stanimirovic DB, Friedman A (2012). Pathophysiology of the neurovascular unit: disease cause or consequence?. J Cereb Blood Flow Metab Off J Int Soc Cereb Blood Flow Metabol.

[253] Stern Y, Gurland B, Tatemichi TK, Tang MX, Wilder D, Mayeux R (1994). Influence of education and occupation on the incidence of Alzheimer's disease. JAMA.

[254] Stewart R (2002). Vascular dementia: a diagnosis running out of time. Br J Psychiatry.

[255] Stys PK, Waxman SG, Ransom BR (1992). Ionic mechanisms of anoxic injury in mammalian CNS white matter: role of Na^+^ channels and Na^(+)^–Ca^2+^ exchanger. J Neurosci.

[256] Sudduth TL, Powell DK, Smith CD, Greenstein A, Wilcock DM (2013). Induction of hyperhomocysteinemia models vascular dementia by induction of cerebral microhemorrhages and neuroinflammation. J Cereb Blood Flow Metab.

[257] Sun Z-K, Ma XR, Jia YJ, Liu YR, Zhang JW, Zhang BA (2014). Effects of resveratrol on apoptosis in a rat model of vascular dementia. Exp Ther Med.

[258] Sweeney MD, Montagne A, Sagare AP, Nation DA, Schneider LS, Chui HC, Harrington MG, Pa J, Law M, Wang DJJ (2019). Vascular dysfunction—the disregarded partner of Alzheimer's disease. Alzheimers Dement.

[259] Takeshita Y, Ransohoff RM (2012). Inflammatory cell trafficking across the blood-brain barrier: chemokine regulation and in vitro models. Immunol Rev.

[260] Takeuchi O, Akira S (2010). Pattern recognition receptors and inflammation. Cell.

[261] Tampi RR, Tampi DJ, Balachandran S, Srinivasan S (2016). Antipsychotic use in dementia: a systematic review of benefits and risks from meta-analyses. Ther Adv Chronic Dis.

[262] Tan R, Traylor M, Rutten-Jacobs L, Markus H (2017). New insights into mechanisms of small vessel disease stroke from genetics. Clin Sci.

[263] Tanaka K-I, Kawahara M (2017). Copper enhances zinc-induced neurotoxicity and the endoplasmic reticulum stress response in a neuronal model of vascular dementia. Front Neurosci.

[264] Tang D, Kang R, Berghe TV, Vandenabeele P, Kroemer G (2019). The molecular machinery of regulated cell death. Cell Res.

[265] Tanovic A, Alfaro V (2006). Glutamate-related excitotoxicity neuroprotection with memantine, an uncompetitive antagonist of NMDA-glutamate receptor, in Alzheimer’s disease and vascular dementia. Rev Neurol.

[266] Tarumi T, Shah F, Tanaka H, Haley AP (2011). Association between central elastic artery stiffness and cerebral perfusion in deep subcortical gray and white matter. Am J Hypertens.

[267] Tasci I, Safer U, Naharci MI, Gezer M, Demir O, Bozoglu E, Doruk H (2018). Undetected peripheral arterial disease among older adults with Alzheimer's disease and other dementias. Am J Alzheimers Dis Other Demen.

[268] Tham W, Auchus AP, Thong M, Goh M-L, Chang H-M, Wong M-C, Chen CPLH (2002). Progression of cognitive impairment after stroke: one year results from a longitudinal study of Singaporean stroke patients. J Neurol Sci.

[269] Tomimoto H, Akiguchi I, Suenaga T, Nishimura M, Wakita H, Nakamura S, Kimura J (1996). Alterations of the blood–brain barrier and glial cells in white-matter lesions in cerebrovascular and Alzheimer's disease patients. Stroke.

[270] Topiwala A, Allan CL, Valkanova V, Zsoldos E, Filippini N, Sexton C, Mahmood A, Fooks P, Singh-Manoux A, Mackay CE (2017). Moderate alcohol consumption as risk factor for adverse brain outcomes and cognitive decline: longitudinal cohort study. BMJ.

[271] Toth P, Tarantini S, Csiszar A, Ungvari Z (2017). Functional vascular contributions to cognitive impairment and dementia: mechanisms and consequences of cerebral autoregulatory dysfunction, endothelial impairment, and neurovascular uncoupling in aging. Am J Physiol Heart Circ Physiol.

[272] Toyama K, Koibuchi N, Uekawa K, Hasegawa Y, Kataoka K, Katayama T, Sueta D, Ma MJ, Nakagawa T, Yasuda O (2014). Apoptosis signal-regulating kinase 1 is a novel target molecule for cognitive impairment induced by chronic cerebral hypoperfusion. Arterioscler Thromb Vasc Biol.

[273] Tripolt NJ, Stekovic S, Aberer F, Url J, Pferschy PN, Schröder S, Verheyen N, Schmidt A, Kolesnik E, Narath SH (2018). Intermittent fasting (alternate day fasting) in healthy, non-obese adults: protocol for a cohort trial with an embedded randomized controlled pilot trial. Adv Ther.

[274] Troen AM, Shea-Budgell M, Shukitt-Hale B, Smith DE, Selhub J, Rosenberg IH (2008). B-vitamin deficiency causes hyperhomocysteinemia and vascular cognitive impairment in mice. Proc Natl Acad Sci U S A.

[275] Troncoso JC, Zonderman AB, Resnick SM, Crain B, Pletnikova O, O'Brien RJ (2008). Effect of infarcts on dementia in the Baltimore longitudinal study of aging. Ann Neurol.

[276] Truettner JS, Alonso OF, Dietrich WD (2005). Influence of therapeutic hypothermia on matrix metalloproteinase activity after traumatic brain injury in rats. J Cereb Blood Flow Metab.

[277] Ueno M, Tomimoto H, Akiguchi I, Wakita H, Sakamoto H (2016). Blood–brain barrier disruption in white matter lesions in a rat model of chronic cerebral hypoperfusion. J Cereb Blood Flow Metab.

[278] Ungvari Z, Kaley G, de Cabo R, Sonntag WE, Csiszar A (2010). Mechanisms of vascular aging: new perspectives. J Gerontol A Biol Sci Med Sci.

[279] Utech M, Mennigen R, Bruewer M (2010). Endocytosis and recycling of tight junction proteins in inflammation. J Biomed Biotechnol.

[280] van de Rest O, Berendsen AA, Haveman-Nies A, de Groot LC (2015). Dietary patterns, cognitive decline, and dementia: a systematic review. Adv Nutr.

[281] van der Flier WM, Skoog I, Schneider JA, Pantoni L, Mok V, Chen CLH, Scheltens P (2018). Vascular cognitive impairment. Nat Rev Dis Primers.

[282] Varatharaj A, Galea I (2017). The blood–brain barrier in systemic inflammation. Brain Behav Immun.

[283] Venkat P, Chopp M, Chen J (2015). Models and mechanisms of vascular dementia. Exp Neurol.

[284] Verbaten MN (2009). Chronic effects of low to moderate alcohol consumption on structural and functional properties of the brain: beneficial or not?. Hum Psychopharmacol.

[285] Verdelho A, Madureira S, Ferro JM, Baezner H, Blahak C, Poggesi A, Hennerici M, Pantoni L, Fazekas F, Scheltens P (2012). Physical activity prevents progression for cognitive impairment and vascular dementia. Stroke.

[286] Vernooij MW, Van der Lugt A, Ikram MA, Wielopolski PA, Vrooman HA, Hofman A, Krestin GP, Breteler MM (2007). Total cerebral blood flow and total brain perfusion in the general population: the Rotterdam scan study. J Cereb Blood Flow Metab.

[287] Wallace DC (2001). A mitochondrial paradigm for degenerative diseases and ageing. Novartis Found Symp.

[288] Wallin A, Kapaki E, Boban M, Engelborghs S, Hermann DM, Huisa B, Jonsson M, Kramberger MG, Lossi L, Malojcic B (2017). Biochemical markers in vascular cognitive impairment associated with subcortical small vessel disease—a consensus report. BMC Neurol.

[289] Wang S, Cao C, Chen Z, Bankaitis V, Tzima E, Sheibani N, Burridge K (2012). Pericytes regulate vascular basement membrane remodeling and govern neutrophil extravasation during inflammation. PLoS ONE.

[290] Wang T, Liu CZ, Yu JC, Jiang W, Han JX (2009). Acupuncture protected cerebral multi-infarction rats from memory impairment by regulating the expression of apoptosis related genes Bcl-2 and Bax in hippocampus. Physiol Behav.

[291] Ward NC, Watts GF, Eckel RH (2019). Statin toxicity. Circ Res.

[292] Wardlaw JM, Doubal F, Armitage P, Chappell F, Carpenter T, Maniega SM, Farrall A, Sudlow C, Dennis M, Dhillon B (2009). Lacunar stroke is associated with diffuse blood–brain barrier dysfunction. Ann Neurol.

[293] Webb AJS, Simoni M, Mazzucco S, Kuker W, Schulz U, Rothwell PM (2012). Increased cerebral arterial pulsatility in patients with leukoaraiosis. Stroke.

[294] Wentzel C, Rockwood K, MacKnight C, Hachinski V, Hogan DB, Feldman H, Østbye T, Wolfson C, Gauthier S, Verreault R (2001). Progression of impairment in patients with vascular cognitive impairment without dementia. Neurology.

[295] Wiysonge CS, Bradley HA, Volmink J, Mayosi BM, Opie LH (2017). Beta-blockers for hypertension. Cochrane Database Syst Rev.

[296] Wu C-x, Liu R, Gao M, Zhao G, Wu S, Wu C-f, Du G-h (2013). Pinocembrin protects brain against ischemia/reperfusion injury by attenuating endoplasmic reticulum stress induced apoptosis. Neurosci Lett.

[297] Wu L-Y, Kan CN, Cheah IK, Chong JR, Xu X, Vrooman H, Hilal S, Venketasubramanian N, Chen CP, Halliwell B (2022). Low plasma ergothioneine predicts cognitive and functional decline in an elderly cohort attending memory clinics. Antioxidants.

[298] Wu LY, Cheah IK, Chong JR, Chai YL, Tan JY, Hilal S, Vrooman H, Chen CP, Halliwell B, Lai MKP (2021). Low plasma ergothioneine levels are associated with neurodegeneration and cerebrovascular disease in dementia. Free Radic Biol Med.

[299] Wu X, Sun J, Li L (2013). Chronic cerebrovascular hypoperfusion affects global DNA methylation and histone acetylation in rat brain. Neurosci Bull.

[300] Xiang C, Wang Y, Zhang H, Han F (2017). The role of endoplasmic reticulum stress in neurodegenerative disease. Apoptosis.

[301] Xiong Z-G, Zhu X-M, Chu X-P, Minami M, Hey J, Wei W-L, MacDonald JF, Wemmie JA, Price MP, Welsh MJ (2004). Neuroprotection in ischemia: blocking calcium-permeable acid-sensing ion channels. Cell.

[302] Xu W, Qiu C, Gatz M, Pedersen NL, Johansson B, Fratiglioni L (2009). Mid- and late-life diabetes in relation to the risk of dementia: a population-based twin study. Diabetes.

[303] Yamada M, Kasagi F, Sasaki H, Masunari N, Mimori Y, Suzuki G (2003). Association between dementia and midlife risk factors: the radiation effects research foundation adult health study. J Am Geriatr Soc.

[304] Yang T, Sun Y, Lu Z, Leak RK, Zhang F (2017). The impact of cerebrovascular aging on vascular cognitive impairment and dementia. Ageing Res Rev.

[305] Yang Y, Jiang G, Zhang P, Fan J (2015). Programmed cell death and its role in inflammation. Mil Med Res.

[306] Yang Z, Zhang N, Shen H, Lin C, Lin L, Yuan B (2014). Microglial activation with reduction in autophagy limits white matter lesions and improves cognitive defects during cerebral hypoperfusion. Curr Neurovasc Res.

[307] Yarlagadda A, Alfson E, Clayton AH (2009). The blood brain barrier and the role of cytokines in neuropsychiatry. Psychiatry.

[308] Yassi N, Hilal S, Xia Y, Lim YY, Watson R, Kuijf H, Fowler C, Yates P, Maruff P, Martins R (2020). Influence of comorbidity of cerebrovascular disease and amyloid-β on Alzheimer’s disease. J Alzheimers Dis.

[309] Yenari MA, Kauppinen TM, Swanson RA (2010). Microglial activation in stroke: therapeutic targets. Neurother J Am Soc Exp NeuroTher.

[310] Yin F, Boveris A, Cadenas E (2014). Mitochondrial energy metabolism and redox signaling in brain aging and neurodegeneration. Antioxid Redox Signal.

[311] Zhang LY, Pan J, Mamtilahun M, Zhu Y, Wang L, Venkatesh A, Shi R, Tu X, Jin K, Wang Y (2020). Microglia exacerbate white matter injury via complement C3/C3aR pathway after hypoperfusion. Theranostics.

[312] Zheng Y, Zhang J, Zhao Y, Zhang Y, Zhang X, Guan J, Liu Y, Fu J (2021). Curcumin protects against cognitive impairments in a rat model of chronic cerebral hypoperfusion combined with diabetes mellitus by suppressing neuroinflammation, apoptosis, and pyroptosis. Int Immunopharmacol.

[313] Zhou Y, Zhang J, Wang L, Chen Y, Wan Y, He Y, Jiang L, Ma J, Liao R, Zhang X (2017). Interleukin-1β impedes oligodendrocyte progenitor cell recruitment and white matter repair following chronic cerebral hypoperfusion. Brain Behav Immun.

[314] Zhu Y, Chai YL, Hilal S, Ikram MK, Venketasubramanian N, Wong B-S, Chen CP, Lai MKP (2017). Serum IL-8 is a marker of white-matter hyperintensities in patients with Alzheimer's disease. Alzheimer's Dement.

[315] Zhu Y, Hilal S, Chai YL, Ikram MK, Venketasubramanian N, Chen CP, Lai MKP (2018). Serum hepatocyte growth factor is associated with small vessel disease in Alzheimer's dementia. Front Aging Neurosci.

[316] Zuccalà G, Onder G, Pedone C, Carosella L, Pahor M, Bernabei R, Cocchi A (2001). Hypotension and cognitive impairment: selective association in patients with heart failure. Neurology.

[317] Zuliani G, Ranzini M, Guerra G, Rossi L, Munari MR, Zurlo A, Volpato S, Atti A, Ble A, Fellin R (2007). Plasma cytokine profile in older subjects with late onset Alzheimer's disease or vascular dementia. J Psychiatr Res.

